# Quarterly Periscope of Practical Medicine: Part I

**Published:** 1827-10

**Authors:** 


### Quarterly Periscope
OF
### PRACTICAL MEDICINE;
BEING
### The Spirit of the Medical Journals,
*Foreign and Domestic;*
WITH COMMENTARIES.

### PART I.

"Ore trahit quodcunque potest, atque addit acervo."

### 1. ANEURISMAL POUCH IN THE HEART.
[M. Cruveilheir.]

In No. 12 of this Journal, page 465-6, we gave some account of Talma's death, and the peculiar disease of the heart under which he laboured—also the case of the late General Kyd, who died of this rare disease, namely, an aneurismal pouch arising from the left ventricle, which ultimately gave way, and caused instant death. M. Berard has recently brought forward two cases of this disease—a third may be found in the Morbid Anatomy of Baillie—and a fourth in the Miscellanea Naturæ Curiosorum. In these cases, as in that of Talma, the fibrous aneurismal pouch arose from the apex of the heart, and was adherent to the pericardium. In a fifth case, reported by Corvisart, the pouch arose from the side of the left ventricle—while, in General Kyd's case, the origin of the pouch was near the basis of the ventricle.

*Case. By M. Cruveilheir.* M. N. aged 77 years, of excellent constitution, and considerable muscular force, summoned M. C. while in a state of suffocation, that took place while in the warm bath. He presented the following symptoms, viz. purple hue of the face—cold perspiration—extreme oppression—anxiety—expectoration of a spumous, sanguinolent character—hard, full pulse, quick and irregular—in short, he had all the symptoms of a severe paroxysm of asthma. On investigation, it was found that he never had experienced a similar attack before, but that ever since 1809 (the time being then 1819) he had complained of dyspnœa and sense of tightness in the region of the heart, for which he consulted the most eminent physicians of Paris, who considered the disease as spasmodic. The patient was directed to keep in the vertical posture before a window—to have pediluvia and frictions applied to the lower extremities, and to take some expectorants. By these means, the sense of suffocation soon went off, and, in a few hours, the patient was restored to his ordinary state of health.

From that period, however, M. N. was quite an altered man. He became irritable, timid, pusillanimous, and haunted with the perpetual dread of death in one of these paroxysms of suffocation. This was the predominant idea. During the day, while surrounded by his children and friends, the terror of death was moderate; but the moment he went to bed, the dread of an attack produced, as if by an electric shock, a sense of constriction about the heart, and a strange sensation in the arm-pits and hams. The precise nature of these sensations he could not clearly describe. He could dilate the thorax, the lungs seemed free, the pulse was habitually hard and full, occasionally intermitting—capillaries of the face injected—digestive functions perfect. He was still solaced by the idea that his disease was merely asthma; but M. Cruveilheir and another physician justly apprehended that they had a more serious malady to combat—namely, an organic disease of the heart. The stethoscope had not yet been discovered; but the fulness of the pulse repelled the idea of there being any contraction of the ventricular orifices. Still there was no doubt entertained of the existence of some organic lesion of the heart. Various means were employed, in the way of hygiene as well as medicine, but not apparently with much effect. During the eight months that intervened between this attack and his death, the patient had several slight accessions of suffocation, which generally came on in the mornings, with a wheezing respiration, hard and irregular pulse, frothy and sanguineous expectoration. The fresh air, horizontal posture, and pediluvia, were generally sufficient to dissipate these attacks.

Early in July, 1820, M. N. was seized with pulmonary catarrh, which was attended with much oppression of the chest, and sanguinolent expectoration. M. Cruveilheir was called to him on the 12th. Nothing particular was done at that time. On the 15th, he was again summoned in great haste. The gentleman had gone to bed in very good spirits, and slept well all night. In the morning, a severe paroxysm of suffocation came on, and carried him off, his intellectual functions remaining unimpaired till the last.

Dissection. The lungs adhered pretty generally, were crepitant, but considerably infiltrated with frothy serum, and the ultimate branches of the bronchia were red. There were four or five ounces of serum in the left cavity of the thorax. The pericardium adhered to the heart by some filaments, especially on the left side. These being torn through, a small tumour, the size of a walnut, presented itself, standing out from the left ventricle of the heart. It adhered to the pericardium. The parietes of the tumour were very resistent. The left ventricle was hypertrophied. Between two of the columnæ carneæ, there was seen a small opening, which would admit the point of the finger, leading to the tumour or pouch above-described. The parietes of this pouch were found to be cartilaginous and osseous, and lined with a fibrinous concretion. There were some calcareous concretions on the mitral valve. The coronary arteries were completely ossified, and the root of the aorta was studded with calcareous spots.

M. Cruveilheir observes that—"here is another instance of asthma *symptomatic* of disease of the heart." It is evident that the patient died of the former, and not of the latter complaint. This is the case in a great number of chronic diseases, where the patient is often cut off by the complication or consequence of the malady, rather than by the original malady itself.

"Is asthma, then, *always* a symptomatic disease? I believe, with M. Rostan, that it is generally such in *old people:*—But I admit, with nosographical writers, a primitive idiopathic asthma; and I found this belief on a multitude of cases, in which there was no evidence, during the intervals, of any organic or functional disorder in the circulation or respiration. I may cite the case of a young man, who fell a victim to the fourth paroxysm of asthma. Several large bleedings were practised, and seemed to accelerate, if not cause the death of the patient. On dissection, I could find no trace of alteration of structure in any of the organs in the chest." In a note, M. Cruveilheir remarks that he has seen bleeding relieve, at one time, the paroxysm of asthma—and at other times, even in the same person, it has aggravated all the symptoms. No medicine has appeared to our author so beneficial in the asthmatic paroxysms as large doses of the gum ammoniacum. He thinks that the disease under consideration consists in a spasmodic constriction of the muscular fibres surrounding the various branches of the bronchia—which constriction is sometimes idiopathic—sometimes symptomatic.

In respect to the aneurismal pouch observed in the above, and in a few other cases, the disease is evidently of the same nature as real aneurism in the arteries of the body. It thus fills up a lacuna in the history of aneurism generally, by shewing that the heart itself is subject to the disease, in the strict meaning of the word.

---

### 2. NEURALGIA CURED BY CARBONATE OF IRON.

We are given to understand that Mr. Hutchinson, who first brought this important remedy into use, does not consider himself well treated by the Editors of medical journals, including ourselves, for not alluding to the discoverer, when they relate cases in which the carbonate of iron has been used. We had given such copious analyses of Mr. Hutchinson's publications when they appeared, that we did not imagine any of our readers could, for a moment, forget, that to him was due the honor of the discovery. However, we give this notice in order to assure him and them that *we* never had any intention of depriving the proper person of all honor due. We moreover state it as our opinion, that the introduction of the carbonate of iron in neuralgic affections was a most valuable measure, and highly deserving the thanks of the profession. For let it never be forgotten, that each individual who successfully employs the discovery of *another*, acquires reputation for *himself*, since his patient attributes to him the merit of the cure.

Some cases have recently been communicated to Mr. Hutchinson by Dr. Darwall and Mr. Wickenden, all exemplifying the efficacy of the carbonate of iron in neuralgic affections.

1. The first case was that of a lady 64 years of age, who, for two years, had been afflicted with severe pain in her face, apparently in the ophthalmic branch of the fifth pair of nerves. In June, 1823, the patient experienced a sudden augmentation of the malady, and calomel purges, leeches, opium, &c. were prescribed without relief. The subcarbonate of iron was then given, in drachm doses, every six hours, and the patient was completely relieved after the sixth dose. She continued well.

The pain in this lady's case was so excruciating in the right suborbital nerve that she sometimes went for weeks together without daring to wash her face.

2. The second case also was under the care of Mr. Wickenden. The pain was seated in the upper lip, accompanied by convulsive twitches. Drachm doses of the specific soon cured the disease.

3. We shall only allude to one more case, communicated by Dr. Darwall. The patient was a young woman, who was affected with severe neuralgic pains in the face, of an intermittent character, and confined exactly to one half of the face and neck. Her health was otherwise good, but her tongue was furred, and she was subject to indigestion. After evacuating the bowels, she took a scruple of the carbonate of iron twice a day, for a week, when she reported an absence of the pain for three days. She omitted the medicine, and the pain returned. The iron was therefore resumed, and continued better than a fortnight, when a cure was effected.

A second case is related by Dr. Darwall, which, as far as one case goes, would lead us to hope that this remedy may prove useful in more than neuralgic or periodical complaints. A gentleman was affected with syphilitic pains, coming on with great severity in the night, particularly affecting one knee, and accompanied by swelling both of the knees and ankles. Half a drachm of the subcarbonate of iron was ordered three times a day. After the second day, the pains ceased, sleep returned, and he complained only of weakness.

### 3. ON THE PASSAGE OF SUBSTANCES INTO THE URINE.

[By Dr. Whoeler. Zeitschrift fur Physiologie von Tiedman.]

This indefatigable physician has made a great number of experiments, with the view of determining what substances pass from the stomach or other parts of man and animals into the urinary secretion—and what we are to infer from this phenomenon? The faculty of medicine, of Heidelberg, decreed the prize to Dr. Whoeler, one of the most distinguished students of that school. We are sorry that our limits prevent us from giving the details of these experiments—we can only afford space for the results. The greater number of these experiments were made on dogs, to which animals the various substances were given in a little paste when their stomachs were empty. They were carefully watched after the ingestion of the experimental matters, and afterwards killed by prussic acid, as the most sudden and the least cruel mode of destroying life. Dr. W. rejected the plan of tying the œsophagus to prevent vomiting, in these animals; justly considering that such an operation must make an impression on the nervous system of the animal that would be very likely to derange the various secretory and excretory functions, and thus vitiate the conclusions. This circumstance has, unfortunately, been too much overlooked by experimental physiologists, and the deception, we fear, has been great in consequence. As it was found to be impossible to introduce a catheter into the bladders of dogs, it was a fortunate thing for the experimenter that he met with a dog who could be made, at any time, to evacuate the bladder by means of threats. This animal served for a great number of experiments.

### I. Physiological Deductions.

It results from the numerous experiments, detailed in the Essay, respecting the substances which pass into the urine, that the following deductions are legitimate:—

*A.* Iron, lead, alcohol, sulphuric ether, camphor, animal oil of Dippæ, musk, and the colouring matter of cochineal, twinsole, vegetable green, and alkanet root, do not pass into the urine. Neither is the quantity of carbonic acid in the urine augmented by the ingurgitation of liquids containing much of that acid.

*B.* We find in the urine, but in a decomposed state, the following substances:—hydro-perferro-cyanate of potass (transformed into hydro-protoferro-cyanate)—the combinations of potassa and soda with the tartaric, malic and acetic acids (in the form of alkaline carbonates)—and the hydro-sulphate of potass, (generally in the form of sulphate of potass.)

*C.* The substances which form new combinations with certain matters of the animal body, and which are secreted in this state by the kidneys are:—sulphur, which passes in the urine, in the state of sulphuric acid, and hydro-sulphuric acid—iodine, evacuated in the form of hydriodate—the oxalic, tartaric, gallic, succinic, and benzoic acids, which are found in the urine combined with an alkali.

*D.* The substances which pass into the urine unchanged are as follow:—the carbonate, chlorate, nitrate, and the hydro-chlorate of potass (the latter, however, more frequently decomposed,) hydro-protoferro-cyanate of potass, subcarbonate of soda, hydro-chlorate of barytes, silicate of potass, tartrate of nickel and of potass;—many of the colouring principles, as of indigo solved in sulphuric acid, of gamboge, rhubarb, logwood, beetroot, black cherries, &c.—many of the odorous principles, (partly modified in odour,) as oil of turpentine, juniper berries, valerian, assafœtida, game, castor, saffron, opium, &c—the narcotic principle of agaricus muscarius, (of Kamschatka)—and, in sickness, oil.

*Notes ad A.* The causes which prevent some substances (*A*) from passing into the urine, may probably be the following:—1. Certain matters are so changed by digestion and chylification, as to be no longer appreciable in the urine. This may easily be conceived of many of the colouring and odoriferous principles. 2. Other substances, as being more congenial to the animal organism, may be completely assimilated, and entirely employed in nutrition. 3. Others, again, may probably be expelled from the system by other channels than the kidneys, as, for instance, camphor and other odorant principles, which may escape by perspiration and pulmonary exhalation. 4. It is probable that some substances, when arrived in the intestinal canal, change into an insoluble state, and become incapable of absorption—or they may so act, by virtue of their astringent qualities, on the mouths of the absorbent vessels, as to indispose these vessels to take them up. This is probably the case with tannin, and the salts of lead, iron, and other metals.

*Ad B.* The circumstance of some substances passing into the urine in a decomposed state, may be attributed to two causes diametrically opposite. Thus the transformation of hydro-perferro-cyanate of potass into hydro-protoferro-cyanate, may depend on a deoxidation by some animal substance—whilst, on the other hand, the passage of hydro-sulphate of potass into the state of simple sulphate, may be owing to oxigination in the act of respiration.

*Ad D.* In respect to the fact of many substances passing into the urine little or nowise changed, it may be observed that most of these substances are of a diuretic nature, and consequently are *excitants* to the kidneys—hence our author thinks he may safely conclude, that whatever excites the kidneys is secreted there with the urine. It is certain, however, that many things prove diuretic without being secreted in the urine. Affections of the mind, venesection, &c. have frequently the effect of exciting the action of the kidneys. Our author comes to the conclusion, (which indeed is the most ancient doctrine,) that all the proximate principles of the urine pre-exist in the blood. This is the conclusion, also, to which Messrs. Prevost and Damas came, as the result of their experiments.

### II. Therapeutic Deductions.

The strong disposition possessed by the vegetable alkaline salts, to be converted into carbonates, in the animal economy, and to pass off in this form by the kidneys, naturally leads to the conclusion that these salts must prove useful in the internal treatment of uric acid formations and depositions in the kidneys and bladder. The alkaline carbonates have been found by experience useful; and are to be given, but not too long, as they weaken the digestive powers. For these we may often substitute with advantage the supertartrate of potass, the sulphate of potass and of soda, the acetate and citrate of potass, and those fruits which contain vegetable alkaline salts, especially cherries, strawberries, &c. These last are found to render the urine alkaline, and may be taken with less disturbance of the digestive organs than the alkaline carbonates.

Acting on this principle, Professor Chelius prescribed cherries to a man who was passing considerable quantities of gravel in the form of uric acid. The disorder disappeared. When the cherry season was over, he took lemonade and cream of tartar with equal advantage. The author has found the supertartrate of potass extremely useful in the same disease.

---

### 4. DYSENTERY OF MADEIRA.

On this subject Dr. Renton has published a paper in the second volume of the Edinburgh Medico-Chirurgical Transactions. Our notice of it shall be short, as it relates merely to the treatment—a subject about which medical practitioners are now less divided than on most other subjects. The disease appears to be very frequent and very fatal in Madeira, especially in the summer and autumn, when it spares neither age, sex, nor station in society. If taken early the disease is manageable; but if neglected, even for a day or two, it frequently baffles medical skill. Dissection is rarely permitted—but when obtained, ulceration of the mucous membrane, generally of the colon, is almost invariably found. In respect to the treatment, Dr. H. observes, from some years' experience, "that in this most formidable and destructive endemic, mercury given boldly and perseveringly till the mouth becomes decidedly affected, is the remedy chiefly entitled to confidence. So thoroughly has repeated observation convinced me of the comparative insignificance of other modes of treatment, and of the efficacy of this, as well as of the freedom from ill consequences, immediate or remote, attending its employment, that, aware of the danger of trifling with the disease at its commencement, I should consider myself guilty of playing with the life of a fellow creature, in hesitating to use it even in cases where there is merely a threatening of the disease." "The whole secret seems to consist in beginning *early*, and in pursuing *steadily*, the object we have in view, before the continuance of the disease has produced any organic lesion, or that degree of constitutional disturbance which is found in all cases effectually to resist the action of mercury." "Out of the many hundreds of cases which I have seen, and treated, I am not aware of a single instance in which I have been foiled in my endeavours to produce salivation, when the patient has applied for relief at the very outset of the disease, and has attended to the directions given him." Calomel with more or less opium was the most convenient form, giving it every three or four hours until the gums became sore. This happens in 24, 36, or 48 hours, "when, instead of the small and bloody stools, and torturing tenesmus, frequent and copious evacuations, of a highly morbid character, take place; with a most gratifying change in the state and appearance of the patient." The motions next become less frequent, and gradually improve in quality. There is some return of appetite and sleep, and recovery generally follows, without any other remedy. Every case in which our author employed venesection terminated fatally.

We need not touch on any of the cases detailed, as they merely exemplify the foregoing methodus medendi.

We may observe that the treatment of iritis and dysentery, both of them active and dangerous inflammations, by mercury, might convince medical men that the sweeping assertions that mercury is always a powerful stimulant, are erroneous. We would ask such asserters, on what principle they explain the curative process?

### 5. GASTRODYNIA.—M. BARRUT.

In the month of September M. Barrut had occasion to see a young man, 16 years of age, who, for fifteen months previously, had been declining in health. The symptoms were reported to be, painful digestion, weakness in his limbs, disgust towards food, head-aches, cold chills, great coldness of the feet. For some time the youth bore up against the malady, and then applied to a physician, who ordered emetics and drastic purgatives. This treatment aggravated the complaint, and he was soon obliged to keep to his bed. A blister was applied to the epigastrium, without benefit—the physician lost the confidence of the family—and the patient was left to the resources of Nature, without any medicines. The symptoms now became mitigated, and returned to their original condition, above-described—the moral and physical powers of the young man being in a state of the greatest prostration. When M. Barrut visited him, he was in extreme emaciation and hebetude—the pulse quick—face rather flushed—tongue furred, but red at the point and sides—appetite sometimes annihilated, sometimes voracious. He pronounced the disease, in his own mind, to be *chronic gastro-enteritis;* and therefore ordered mucilaginous drinks, and leeches to the epigastrium. The former part of the prescription was followed, but not the latter. In a few days our author called on the patient, and found him much worse. He was in a state of great irritability, and it was then evident that he was kept in a state of irritation by his parents. The system was changed—he was ordered light nourishing animal food, with a moderate allowance of old wine mixed with cold water—to be clothed in flannel—and to take regularly increased exercise of all kinds, carried to the extent of moderate fatigue. M. Barrut kept his eye on the patient for a fortnight, and directed all his regimen and exercise. The change for the better was surprising. In a short time he was entirely well, and grew up a fine stout young man. It is to be remarked that, for some days after commencing the animal food, the patient suffered severely in his digestive organs; but the inconvenience gradually subsided as his strength increased.

M. Barrut candidly confesses that he came to this case with a prejudiced mind—and that he saw, or fancied he saw, nothing but a "superb" *gastro-enterite.* Had the patient gone on with leeches and gum water, he would probably have died. This treatment was as injudicious as the original one by emetics and drastic purgatives. The mental irritation had a great share in the gastric affection;—when this was withdrawn, and light animal food with generous wine prescribed, the cure was soon effected.

### 6. ASCITES WITH PREGNANCY.

We have brought forward several cases in this Journal, where the operation of paracentesis was successfully performed during pregnancy. The following is another addition to the list, from the practice of Mr. James Russel, as recorded in the Medical and Physical Journal for May.

Mrs. Kelly, in the sixth month of pregnancy, was visited by Mr. R. who found the abdomen greatly distended—the legs œdematous—and the rest of the body emaciated. There were present also dyspnœa and constipation of the bowels. She was bled, and took calomel and jalap, which measures relieved the urgent symptoms, and Mr. R. was not again called for a week or ten days; when dyspnœa and painful tension of the abdomen were complained of. She was again bled and purged, with relief; but, in ten days more, the dyspnœa and cough were so alarming, that Mr. R. decided on tapping. The patient was now in the seventh month of pregnancy. The trocar was introduced two inches below the umbilicus, and twenty-three quarts of fluid were drawn off. The cough ceased, the respiration became free, and the tumefaction of the labia pudendi and legs soon subsided. She went on improving for more than a month, when labour came on, and she was quickly delivered of a feeble and emaciated child, which only lived ten days. In two months, the dropsical accumulation had again got to some size, and required the operation of paracentesis, which evacuated thirty quarts of water. The dropsy was a third time returning, when Mr. R. lost sight of the patient.

This, and other cases on record, prove the safety, and even the propriety of the operation during pregnancy. In general, the labour comes on soon after paracentesis; but in this instance it was otherwise.

### 7. SALIVATION FATAL.

Mercurial salivation, especially in adults, is so rarely fatal, that the following case deserves record—more as a curiosity, however, than as holding out any rule of conduct by which we can be guided in future; for here there was no misconduct on the part of the medical practitioner, nor any apparent peculiarity in the constitution of the patient, to contra-indicate the use of mercury.

*Case.* A soldier, 22 years of age, was admitted into a Regimental Hospital on the 22d February, for the treatment of chancres situated at the base of the glans penis. Half a drachm of mercurial ointment was rubbed in every second day, and a grain and a half of the oxymuriate of mercury being divided into four doses, he was ordered to take one dose on alternate days. On the 9th day, having made five frictions, and taken the above quantity of oxymuriate, salivation appeared, and the mercury was discontinued. The ptyalism ran high; and low diet, cooling aperients, gargles, and various other means were resorted to, but without the least effect in checking the salivation. The tongue became greatly swelled and indurated—the cheeks ulcerated—the gums were destroyed, and the teeth loosened. Deglutition was extremely difficult—and the nights sleepless. The venereal ulcers rapidly disappeared. Leeches were applied in succession to the face and neck, and opiates were given, with the view of procuring some sleep. But nothing arrested the progress of the ptyalism, and its devastations on the mouth, tongue, and throat. On the 5th April, incisions were made into the tongue, with the hope of relieving the swelling of that organ; but little good effect resulted. By the 18th of April the salivation began to diminish, and ceased entirely by the end of the month. The swelling of the tongue disappeared, and the ulcers of the mouth healed. But the man continued pale and emaciated, complaining of sense of heat in the epigastrium, and having cough, without expectoration. Early in May, expectoration appeared, and was accompanied by diarrhœa. He soon began to throw up purulent matter, and he died of rapid pulmonary consumption on the 14th May, not quite three months from the commencement of the mercurial treatment.

On dissection, the mouth, and parts contained in it, were found perfectly healthy. In the left lung a large abscess presented itself, full of fetid matter, a quantity of which was extravasated into the bag of the pleura on that side. There was nothing particular in any other part of the body. *Journ. Complementaire, Fevrier.*

The above is a melancholy case; but is it to prevent our administering mercury in a similar case of syphilis? Two or three drachms of mercurial ointment, and a grain and a half of oxymuriate of mercury in nine days, are not quantities of the mineral from which any danger can be apprehended, under ordinary circumstances—and the extraordinary circumstances, that is, the idiosyncrasies of constitution to which mercury may be poisonous, are utterly incognizable, till the mischief is done!

---

### 8. SECTION OR LIGATURE OF THE PNEUMO-GASTRIC NERVES.

[Professor Mayer, of Bonn.]

In a series of experiments, the above-mentioned Professor endeavoured to ascertain the cause of death from section or ligature of the pneumogastric nerves. He chose the latter mode of experimenting in general, because the death was then more gradual, and the effects of the ligature more easily ascertained. By these experiments, he thinks he has refuted the conclusions to which Dr. Philip came, respecting the influence of these nerves on digestion. But the Professor is evidently unacquainted with the discovery which Dr. P. made, and which we ourselves witnessed, that mere section of the nerves, without separating considerably their cut extremities, was insufficient for the interruption of digestion. The nervous influence was still transmitted along the divided nerve, if the extremities were left in contact, or nearly in contact. The mere ligature, then, of the par vagum, would be attended with a still greater vitiation of the experiment, as far as digestion was concerned. It is interesting, however, to observe the phenomena produced by the interruption of nervous energy in other organs and parts, besides the stomach.

The following are the results of numerous experiments:—

1. Professor Mayer always observed that when death took place some considerable time after the ligature of the nerves, there were found in the heart and lungs those white coagulations formerly called polypi. These concretions occupied the cavities of the heart, and also the arteries and veins of the lungs. Willis, Baglivi, and others, indeed, had noticed the same phenomenon, but did not attach sufficient importance to it. These coagulations are soft, and of a black colour, if the death take place suddenly after the operation—that is, within ten or twenty hours. If death do not take place till after 40 hours, the concretions are white and like polypi. It is to these formations that the Professor attributes the death of the animal, by interrupting the circulation. The nervous influence being withdrawn, or greatly diminished, the blood loses its fluidity, and begins to coagulate and become decomposed, as if it were out of the body. Hence we may conclude that the fluidity of the blood is owing to nervous influence. These facts, Professor M. thinks, may tend to throw some light on the nature of asthma, which is probably owing to a temporary diminution of nervous energy, and consequently to an incrassation of the blood in the lesser circulation.

2. Another frequent, but not constant accident, which takes place after the ligature, is the entrance of alimentary matters rejected from the stomach into the trachea, from the relaxed state of the glottis. This accident generally destroys life very quickly. In these experiments an antiperistaltic movement takes place in the stomach, by which the food is thrown up into the pharynx, and there enters the aerial passages.

3. In some rare cases there takes place an infiltration of air under the mucous membrane of the bronchia, from a rupture of that membrane, and an emphysema is the consequence. This accident terminates the life of the animal, by suffocation.

4. A remarkable and constant phenomenon which was observed, consists in an antagonism (as it may be called) between the pulse and the respiration. The activity of the heart is often doubled—while the respiration is rendered much slower than natural. Notwithstanding this slowness of the breathing, the temperature of the animals remained nearly stationary.

5. In these experiments, the gastric digestion was not interrupted, except where the antiperistaltic action of the organ took place, and consequently where the food was ejected. These experiments, then, were considered as subversive of those of Dr. Philip; but, for the reasons already stated, they do not at all affect the results of the English Physiologist.—*Zeitschrift fur Physiol.*

---

### 9. GLOSSITIS TREATED BY INCISIONS.

[Mr. Martin. Ed. Journal.]

A stout man, aged 35 years, had been employed in building a wall, on the 7th February; and, in the evening, felt acute pain in his tongue, at first confined to a point, but soon extending over the whole surface.

The lingual arteries throbbed violently, and the saliva was tenacious—there was restlessness, with some fever and head-ache. The tongue increased in size till it protruded from the mouth and separated the jaws. On the 9th he consulted Mr. Martin. On examination, the organ felt smooth and hard, with a thick coating of viscid saliva—countenance anxious—fever. The patient was bled from the arm to 30 ounces, with some relief. The venesection was repeated to the same extent in an hour afterwards—and then he could articulate somewhat intelligibly. In three hours more the lingual tumefaction had increased—respiration through the mouth was impracticable, and that through the nostrils was difficult, pulse fluttering, breath offensive—in short, suffocation was threatened.

A deep incision was made in the most prominent part of the right side of the tongue, from whence issued a quantity of blood and pus, with evident relief. Two other incisions were made when the first ceased to discharge blood. The bleeding was promoted by gargles. In a quarter of an hour after the first incision, the patient could articulate distinctly—the respiration became free, and the countenance cleared up. Next day he might be said to be well.

---

### 11 PROTEIFORM MALADY.

The following authentic case of a disorder extremely prevalent in this country, but which, under various disguises, passes for other complaints of a very different character, is recommended to the inexperienced practitioner, as an instance that may awaken his attention to a most fruitful source of deception in the practice of his art.

*Case.* Mr. B. aged about 31 years, of a robust constitution, and, from infancy, (with the exception of one or two bilious attacks,) in the enjoyment of uninterrupted health, had contracted a gonorrhœa, which, though unattended by any aggravated symptoms, had continued to be troublesome for two or three months. He consulted a surgeon in the country where he happened then to be, by whose advice he used astringent injections; but still continuing to feel irritation in the urethra, with a slight discharge, this person suggested to him the probability of a stricture, and recommended the introduction of a bougie, which was accordingly done. The operation was attended with such sudden and violent pain, that he, Mr. B. was with difficulty kept from fainting. The pain and irritation thus caused continued to increase till the following day, when inflammation of the testicle came on. An aperient was immediately given, with doses of antimonial powder every four hours, and poultices were applied. A general and extraordinary disturbance of the system followed. The tongue became thickly furred, with much fever, great anxiety, and restlessness, and also slight delirium, and obstinate constipation of the bowels. Evacuations were, however, at length obtained, by means of powerful aperients, and enemas, and the antimonial powders were continued. Within 48 hours after this attack, Mr. B. was in such a state of exhaustion, as to be unable to raise himself in his bed without assistance, experiencing extreme disorder and distension of the stomach and bowels, and the most distressing hysterical symptoms, the organs of speech being affected in a very remarkable manner. His condition appeared so alarming, that a physician was called, who prescribed such remedies as alleviated the above-mentioned symptoms, and the antimonial powders were discontinued. The local inflammation gradually subsided, and Mr. B. was slowly recovering the effects of his late attack, when the surgeon who had attended him suggested the probability of a connexion between gonorrhœa and lues. With a view to complete the cure, (as he said,) and, to guard against any lurking taint, he recommended a slight course of mercury. The mercury was persisted in till the metallic taste was perceptible, when it was discontinued. Pain in the testicle and adjoining parts continued still to be felt, extending down the thighs and legs, and also severely affecting both the hip-joints. The warm-bath was then recommended, "to get the mercury out of the system." This state continued for about three weeks, when Mr. B. was suddenly attacked by a most severe pain, extending from one eye to the other, across the upper part of the bridge of the nose, accompanied by a very distressing and copious perspiration. The perspirations becoming frequent, the medical attendant prescribed sulphate of quinine, which was accordingly taken for a short time. About a month after the attack of pain in the bridge of the nose, the pain that had hitherto been felt in the thighs, legs, and hip-joints, suddenly ceased, and about five days elapsed without pain of any kind; when the patient was again suddenly seized with very violent head-ache, pain in the bridge of the nose as before, and in the right shin bone. Mr. B. being in the neighbourhood of *****, went there for the purpose of consulting Mr. *****. This was in the beginning of October, 1826. On the road to *****, his face was exposed to a very cold wind, and, the same night, he was seized with most acute pains in the nose and shins, the former being much swollen on the following day. Mr. B. considered that this attack might be the effect of cold caught after taking mercury. On the second night, Mr. B. was seized with violent spasmodic twitchings in the thighs and legs, which lasted the greater part of the night, but were at length relieved by perspiration. The pains in the head, nose, and shins continued for three months, becoming gradually less, but attended by clammy sweats of a very distressing nature, which generally came on in the morning, on waking. About this time, the pain in the nose frequently extended to the two front teeth. Having found that exposure to the cold air invariably brought on severe pains in the nose and face, Mr. B. was obliged to confine himself altogether to the house. Towards the latter end of this period, the stomach and bowels were much distended with flatulence, attended by much pain, and dyspnœa. Mr. B. continued under the care of Mr. *****, who seemed to confine himself to watching the symptoms, giving him small doses of rhubarb or Epsom salts to keep the bowels open, as they were in a very inactive state.

Early in the month of January, of the present year, about six o'clock one evening, Mr. B.'s tongue was suddenly seized with a spasmodic contraction, the point being turned downwards towards the root; and, as the evening advanced, he experienced increasing nervous irritation, the tongue becoming quite fixed, and the voice inarticulate. On Mr. B.'s arrival, he ordered him to bed, and gave him a sudorific, which acted powerfully. Hysterical symptoms, with great distress and distension of the stomach, came on, with alternate fits of delirium and deep depression of spirits. He continued in this state throughout the night. It should be remarked that, for some days before this attack, the bowels had been in a very costive state; the evacuations, when they occurred, being small and extremely hard. Mr. *****, on the night of this attack, had given him some pills of colocynth, which not operating, he prescribed in the morning a very strong dose of salts, senna, and magnesia, which produced, in the course of the day and following night, a dozen or more of copious, very dark coloured, and excessively fetid stools. The effect of this was to mitigate the general symptoms, but the perspirations continued, producing great distress, and the tongue was still tied down. A very singular effect was invariably produced on the voice, when any violent access of perspiration came on, and then the hysterical symptoms returned with aggravation. Dr. ****, of ****, was called in, and, in consultation with Mr. *****, the complaint appears to have been, for the first time, considered as a disorder of the digestive organs. In order to check the perspirations, nitric acid was given in the decoction of sarsaparilla, and the senna, salts, &c. were taken every morning at first, and afterwards every second morning. Under this treatment, Mr. B. continued for about six weeks. It may be observed that, for some weeks, both before and after this attack, the least attempt to read or write brought on immediately the most violent head-aches. The tongue, however, had become gradually unbound towards the end of the six weeks; but the head-aches had got more severe, and, finally, the stomach revolted at the nitric acid. This circumstance, and the quantity of lithic acid deposited in the urine, induced Dr. **** to substitute carbonate of soda in the decoction of sarsaparilla, at the same time continuing the purgation as before. At the expiration of a fortnight, the soda, disagreeing with the stomach, was also discontinued; and the compound colocynth pill given in lieu of the salts and senna. But, as the doses were moderate, the bowels again became constipated, and continued so for two or three days, at the expiration of which, a sudden and violent salivation came on, quickly producing extreme debility and depression of spirits. The flow of saliva amounted, for the first five or six days, to at least a pint a day. Great relief to the head and tongue immediately ensued, which caused it at first to be considered as a salutary effort of nature; but, on its continuing and producing very distressing effects, and Mr. B. becoming much emaciated and weakened, Dr. **** tried an astringent gargle, which had no effect; and, the debility increasing, Mr. B. left *****, about the latter end of March, for Torquay, in the hope that change of air, &c. might benefit him. There he remained till within a week, (27th June.) He has persisted in the use of the colocynth pill up to the present time; the spitting still continues, with little intermission; and although he has availed himself of every possible means of obtaining benefit from air and moderate exercise, chiefly in a carriage, he gains neither flesh nor strength.

Such is the history of this remarkable (though by no means uncommon) case, which the patient presented to the reporter, on the 27th June last. Mr. B. is a gentleman of very active habits, when in health, and had recently spent some time among the blue mountains in Jamaica, exploring the geology of those interesting regions. The history of the case and the appearance of the patient left no doubt in the mind, as to the true nature of the malady; and the first question which the reporter asked Mr. B. was this:—"did you experience any *mental* affliction a short time before this complaint commenced?" The gentleman seemed surprised—and, after a pause, he observed that he *had* experienced some most distressing *moral* afflictions, previously to the commencement of the train of *physical* phenomena above described. This unravelled the mystery. The nervous system had received a shock—the digestive organs became deranged in function—and then the whole of the nervous system was soon rendered morbidly susceptible to every impression. The Proteian malady, as may be seen, has already assumed many forms, though as to nature essentially the same in all. It will probably assume many more, unless the chain of morbid sympathies and associations be broken by the plan which has been laid down for the invalid. This plan is a tour among the mountains of Switzerland, with rigid temperance. It may be observed that such is the state of morbid sensibility in his stomach and bowels that he dares scarcely take as much food as is sufficient to support nature. The consequence is, extreme emaciation. He has been advised by the reporter to gradually increase the quantity and vary the quality of his food and drink, as he proceeds on his journey, increasing also his corporeal exercise in proportion as his muscular power returns. He is desired to take no other medicine than just as much of the compound colocynth extract, with rhubarb and ipecacuan, as will keep the bowels in daily action. Under this plan it is confidently hoped that recovery will take place, as, on accurate examination, there is no evidence of any organic disease in any part of the body.

---

### II. TRANSFUSION OF BLOOD.

[Mr. Fox. Med. and Phys. Journal.]

The following fact is added to those now accumulating in respect to this interesting subject.

A female, aged 30, having advanced to the sixth month of pregnancy, was suddenly seized with labour pains which terminated in expulsion of the fœtus. Considerable hæmorrhage ensued, and Mr. Fox's attendance was demanded. He found the patient greatly exhausted by loss of blood, the pulse being feeble, and syncope impending. The placenta not having come away, Mr. F. directed the patient to take laudanum and some stimulants, while cold water was plentifully applied to the lower part of the abdomen. At the same time the hand was introduced into the uterus, where an hour-glass contraction was found, with the greater part of the placenta in the upper portion. The hæmorrhage continued to an alarming extent, but was arrested by irritating with the fingers the internal surface of the uterus, while pressure was made externally. The hæmorrhage controlled, there was still reason to apprehend immediate dissolution from extreme exhaustion—the power of articulation, motion, and deglutition having completely failed. The pulse was imperceptible, and the countenance of the patient wore the aspect of death. A common tea-cupful of blood was injected from the vein of a by-stander, occupying about six minutes. The pulse now became perceptible, and articulation was soon practicable, as well as deglutition. The arterial action and vital energy now returned rapidly, and the recovery was complete.

We have already more than once observed that an unreasonable degree of scepticism obtains respecting the efficacy of transfusion. If the patient dies, the inutility of the measure is pronounced upon without appeal. If the patient recover—Oh! it was by the powers of Nature, and the transfusion had nothing to do with the recovery! Thus the supporters of sanguineous transfusion are very hardly treated, and it becomes absolutely impossible to satisfy the scepticism of their opponents.

---

### 12. LIGATURE OF THE COMMON ILIAC.

[Dr. Mott. New York.]

Professor Mott of New York, has recently performed this operation for the cure of aneurism, which, although, but of ten days standing, occupied the whole extent of the vessel, from within the ligament of Poupart, to some distance above the origin of the internal iliac artery. The tumour was of large size, protruding the belly considerably at the iliac region; the patient suffered excruciating pain, which appeared to increase, as the tumour enlarged. Dr. Mott's incision extended from the external abdominal ring, to one or two inches above the crest of the ilium, dividing the tendon of the external oblique, and cutting through part of the origins of the internal oblique and transversalis. He then cautiously raised the peritoneum with his fingers, and succeeded in detaching it entirely from the tumour and vessels, without doing it the slightest injury.

The artery was then examined, and the aneurismal dilatation was found to cease at about half the distance between the bifurcation of the aorta and the origin of the internal iliac branches. The ligature was passed from the outside of the vessel, by the aid of the excellent instrument recommended by Drs. Parish and Hewson,* carefully avoiding the iliac vein.

* See their valuable paper in the Electic Repertory, on ligature at the subclavian, &c.

The protrusion of the intestines rendered this part of the operation the most difficult. After the ligature was passed around the vessel, the wound was held open in such a manner as to allow the medical gentlemen present to see, and satisfy themselves of the exact situation of the ligature, which was just below the bifurcation of the aorta into the primitive iliacs, and on the side of the sacro-vertebral promontory. The ligature was then drawn tight and secured, the pulsation of the tumour ceased, its size was much diminished, and the patient was relieved from the agonizing pain, previously unremitting.

The wound was lightly dressed and the patient put to bed; the limb of the side operated on, was cold, as might be anticipated; it was wrapped in cotton, and covered up to preserve the temperature, until the circulation should be restored. To the great surprize and gratification of the surgeon, in little more than an hour after the operation, the circulation and temperature were entirely restored, and all fear respecting the supply of blood to the limb, effectually dissipated.

No untoward circumstance has since occurred, now nearly a month, subsequent to the ligature of the artery. The patient has complained of no inconvenience, but a peculiar sensation of fulness or tension in the limb, as if the small vessels had not yet become accustomed to their new office in sustaining the great mass of the circulation for the support of the member. We heartily rejoice at the successful result of this case, for the sake of our fellow creatures, the character of the profession, and the reputation of Professor Mott, who is not more distinguished for admirable skill in operating, and the success of his practice, than for those qualities of head and heart which endear him to such as are under the necessity of submitting to the last and dreadful resort of surgery, the knife.—*Phil. Journ. Med. Science.*

---

### 13. CYNANCHE LARYNGEA—BRONCHOTOMY.

[Dr. Wm. Cullen. Ed. Journal.]

The patient was a female, aged 35 years, to whom Dr. C. was called on the 14th of March, and found her labouring under great difficulty of breathing, especially of inspiration, with flushed face, copious perspiration, prominency and redness of the eyes, anxiety of countenance, paroxysms of dry cough, pulse 120, voice nearly extinct. She pointed to the larynx as the seat of severe pain, and said she felt a load on her chest. The thorax sounded well, but the respiratory murmur was faintly heard with the stethoscope—no râle. The history of the case shewed that the patient had laboured under chronic laryngitis for some time, with great obstruction about the rima glottidis. Purgatives were exhibited, and afterwards opium and ether—blister to the sternum—pediluvia. At one o'clock of the same day, the symptoms had become so exasperated, that bronchotomy was deemed the unicum remedium. It was performed, with the assistance of Dr. Craigie. The opening was made between the cricoid and thyroid cartilages, but as it was found too small to admit a large tube, a part of the thyroid cartilage was slit up. An artery of considerable size was divided, but did not require a ligature, although blood was sucked in at each inspiration. The presence of the tube excited coughing; and a tough expectoration was ejected through the canula. The patient soon breathed easily through the tube, and "the woman said she felt very much relieved." The patient put her finger on the tube when she replied to Dr. C's questions. Without this she could not, of course, articulate. The tube was kept in a fortnight, and then removed, without any inconvenience, and the wound was healed.

Dr. C. is convinced that the operation of bronchotomy was absolutely necessary in the above case, and we are bound to believe it was, on his statement of the symptoms. At the same time we are rather surprised that the tube could be dispensed with at the end of a fortnight, where the disease was originally chronic laryngitis. A sudden attack of acute cynanche might render necessary bronchotomy, and the cause of the operation might be dissipated in the above period; but in chronic cases, there is generally so much change of structure as to require the artificial opening much longer.

Dr. Cullen is decidedly wrong in one physiological remark which he has made. "As soon as an opening is made in the trachea the patient can no longer cough." We can aver in the negative to this. We sat up a whole night with a patient after a tube had been introduced, and the patient coughed out the tube several times in the course of the night. True it is that the patient cannot close the top of the trachea, one of the actions in coughing—but the other and main action, the "violent expiration," can be readily performed—and this is, to all intents and purposes, the phenomenon of coughing. Dr. C. is therefore wrong in saying that a patient with a tube in the trachea or larynx "can only produce a current of air too weak for the purpose of expectoration." We have seen a patient throw out matters through a tube in the trachea, with such force as to project them twelve feet from the bed where he was lying. The patient to which Dr. C. alludes, as having died a few minutes after the operation, in whose trachea some coagulated blood was found, did not die, we are convinced, from the simple inability of expectorating matters after the introduction of the tube. There must have been some other cause. In the very case which Dr. C. has published, there was no expectoration noticed *till after* the tube had been introduced. Indeed it is not a little singular that while Dr. C. maintains that a person with a canula in the trachea cannot cough, he has distinctly stated that—"on the first introduction of the tube *a fit of coughing supervened*, attended, to my great surprise, with expectoration through the canula of a tough semi-transparent mucus, &c." many a theoretical notion has been thus *surprised* by contradicting facts! Nevertheless we give Dr. Cullen all due credit for his operation.

---

### 14. SUCCESSFUL AMPUTATION AT THE HIP-JOINT.

[Dr. V. Mott—of New York.]

Another successful case of this formidable operation is now placed on record to the honour of modern surgery. It is the first instance of the kind which has occurred on the western side of the Atlantic, and entwines an additional wreath of laurel around the brows of Dr. Mott, who is already distinguished as one of the boldest and most adroit surgeons of the United States.

The patient was a fine healthy boy, ten years of age, who broke his thigh, one third of the bone's length from the hip-joint, and seems to have fallen into hands well calculated for giving Dr. Mott the present opportunity of performing a brilliant operation. Be that as it may, the patient was brought to New York in September, 1824, in a condition that left no other chance for life but amputation at the hip-joint. The following is Dr. Mott's concise and modest account of the operation.

"On the 7th of October, 1824, the patient, after having passed a comfortable night, was placed upon the table in order to be operated on. An incision was made over the femoral artery as it emerges from under the femoral arch, and the vessel secured by ligature. While feeling on the outside of the artery for the lesser trochanter, the pulsation of a vessel apparently but little smaller than the femoral artery immediately below the ligature, convinced us that in this case the profunda femoris was given off above the femoral arch, as we occasionally find it. This vessel was taken up.

"Lisfranc's knife was then introduced between the artery and bone, and carried through close by the neck of the femur towards the tuber ischii, thus forming the inner flap. The external flap was formed by cutting from without inwards. The hæmorrhage from the veins and small arteries was considerable when the incisions were made, and numerous vessels were taken up: but comparatively little blood was lost during the operation, and the patient was put to bed shortly after it was completed. After the inner flap was cut, some of the surgical attendants examining the lesser trochanter, pronounced that the head of the bone was *not diseased.* In order to satisfy the doubts expressed, the bone was sawed through the lesser trochanter, when it was found to be of the consistence of cheese, being denuded of periosteum on the outer side up towards the joint, and requiring to be removed, which was afterwards done, as originally contemplated.

"It is scarcely necessary for us to enter into the detail of symptoms and treatment subsequent to the operation, as nothing occurred worthy of note, except various degrees of irritation of the stomach and whole system, previous to the coming away of the ligatures. The treatment consisted in regulating the diet, and administering anodyne and tonic medicines according to circumstances.

"On the 15th of October, eight days from the operation, two-thirds of the stump were healed by the first intention. Between the 17th and 31st of October, all the ligatures, seventeen in number, were removed; and by the 20th of November the whole stump was effectually healed, and the boy had become fat and lusty."—*Philadelphia Journal,* 103.

### 15. CEREBRAL AGENESIS.*

[Salpêtrière.]

The species of paralysis described in this memoir, takes place in the fœtus or in early infancy, and depends on one of two causes—the first being an imperfect development, without alteration of structure, in certain parts of the brain—the second, an alteration of structure, accompanied by a defective development of the part affected and of the neighbouring parts. The whole of these have been designated MALFORMATIONS of the encephalon, and the unfortunate individuals thus circumstanced, have been termed monsters, or monstrosities.

Formerly it was supposed that the nerves took their origin from the brain and spinal marrow; but modern researches have proved that they arise from the different parts to which they were formerly supposed to be sent, and thence concentrate towards the brain and spinal marrow, for the purpose of keeping up the known relations between the sensorium and system at large. It is clearly ascertained that the nerves are formed before the spinal marrow—and that this last is formed before the brain:—in short, the encephalon, strictly speaking, is the last part of the nervous system which is formed by the hand of Nature. It is also a well-known law in the animal economy that, in proportion as the parts are early formed, they are free from defect in organization—hence the frequency of malformations in the brain, as compared with the spinal marrow and nerves. Our author thinks, and we agree with him, that the term *atrophy* is an improper term for imperfect development of the brain. He has therefore adopted the word *agenesis*, which is quite unobjectionable in all respects. Dr. C. has pursued the following order in his researches—1mo. Complete cases, that is, where dissection took place; accompanied by remarks on the correspondence between the encephalon and the other parts of the system. 2ndo. Incomplete cases, (unaccompanied by dissection,) with deductions as to the nature and seat of the cerebral alteration. 3tio. Descriptions of the organic and functional alterations of the members. 4to. The influence of the malformed brain on the other parts of the system and their functions. 5to. Considerations on the epoch at which this cerebral *agenesis* takes place—its comparative frequency, and the parts of the brain most subject to these malformations. 6to. To determine whether paralysis always takes place in the side opposite to the cause in the brain. 7to, and lastly, researches as to the causes, real or presumed, of this cerebral agenesis.

#### COMPLETE CASES.

1. *Case of primitive or congenital Agenesis of the Right Hemisphere.* Mary Masson, aged 59 years, presented the following defective development in the left side of the body. The left arm was much less voluminous than the right, but was nearly of the same length—while

---

* Researches on Cerebral Agenesis and Congenital Paralysis. By J. B. CAZAUVEILH, Intern of the Civil Hospitals of Paris.

the lower left extremity was much shorter, but nearly as thick as the right. All the motions of the left arm were so imperfect, that the member was rendered quite useless. The left lower extremity, from its shortness, caused great lameness, and deformity about the pelvis. The sensibility of the left side, as well as the sight of that eye, was very feeble. The defect of the intellectual faculties was not so striking. She spoke little, and her answers were very laconic. Her temper was mild and unirritable. For a long time this woman had laboured under hypertrophy of the left ventricle of the heart. An acute pneumonia supervened, and she died in hospital under the care of Messrs. Roston and Ferrus.

*Dissection.* The body was opened in the presence of Messrs. Cazaux and Lalesque, when the following appearances presented themselves. The skull was thick, and the dome over the *right* hemisphere was rather flattened. The meninges and vessels of the encephalon were natural. The convolutions of the *right* hemisphere were less developed than those of the *left* side, which last was of much greater volume than the other. The left lateral ventricle was more spacious than the right. The corpora striata did not materially differ in length, but in thickness they did. The right thalamus nervi optici was much less than the other. There was no appreciable difference in the two sides of the cerebellum, tuber annulare, or medulla oblongata et spinalis. The left side of the heart was hypertrophied, and the right lung hepatized.

We forgot to remark that this woman had an impediment of speech; but how far this impediment was connected with the imperfect development of the left side of the brain, it would be difficult to say. There can be little doubt, however, that the atrophy of the members and the defective sensibility of one side of the body were dependent on the congenital agenesis of the brain.

*Case 2. Congenital Agenesis of the Left Hemisphere of the Brain—Paralysis of the Left Side of the Body.* A female, aged 51 years, died in the Salpêtrière in the month of August, 1825, of acute pneumonia, who had been affected with congenital paralysis of the right side of the body. The whole of this side, the face, the mamma, the upper and lower extremities, were much less developed than the corresponding parts of the other side. The arm and leg were not only smaller but shorter than their fellows, and the arm was quite contracted. The motility of these members was much more deteriorated than the sensibility, which last, however, was somewhat obtuse. The intellectual faculties were evidently below par.

*Dissection.* The convolutions on the surface of the *left* hemisphere were much less prominent than those of the right—and indeed the whole of the *former* hemisphere was much smaller than the other. The right hemisphere was nearly half an inch more in diameter than the left. There was no appreciable difference in the ventricles, corpora striata, or thalami optici. The same might be said of the two lobes of the brain, and of the two sides of the medulla oblongata et spinalis. The nerves of the atrophied members appeared as large as those of the sound members.

*Case* 3. Mary M.——, aged 68 years, who had long gained her livelihood by selling milk in the Salpétrière, had been deprived almost entirely of voluntary movements in the upper and lower extremities of the right side since her earliest infancy. The power of the arm had always been extinct; but its volume was as great as that of the other arm. The right lower extremity was considerably undersized in all respects, as well as shortened. Locomotion was performed by means of a crutch, and that very imperfectly. The woman was seized with acute pneumonia, and soon died after her reception in the infirmary of the Salpétrière.

*Dissection.* The cranium, membranes, and vessels, presented nothing particular. The anterior lobe of the *left* hemisphere was less developed than the corresponding one of the other side. In the interior of the former was found a cavity capable of containing an almond, and communicating, by means of a small fistula, with the lateral ventricle of that side. This cavity was lined by a red membrane, with bands crossing from side to side. There was no fluid in the cavity. The two lateral ventricles were of equal dimensions; but there was great inequality in the corpora striata and thalami nervorum opt. The *left* corpus striatum was one-fourth smaller than the *right*, and contained very little medullary substance. Nearly the same difference existed between the two thalami ner. opt. All the other parts of the cerebral and spinal system were equally balanced. The muscles of the paralyzed limbs were small and flabby; while the nerves were larger and of a yellower colour than in the sound limbs. The state of the intellectual faculties were not ascertained by the medical attendants; but they were reported to be below par.

*Case* 4. N——, aged 27 years, died in the Salpétrière on the 7th December, 1825, of chronic gastro-enteritis, on which had supervened acute pneumonia. This woman presented a defective development throughout the whole of the right side of the body. The arm was shrivelled and contracted, and possessed but little motility. The right lower extremity was less than the left, and its motility very imperfect, so that the individual was quite lame. The mouth was habitually drawn to one side, and the pupils of both eyes equally dilated. Sensibility was somewhat defective in the atrophied side. The intellectual faculties were considerably below par. She could not freely express with her tongue the limited range of ideas generated in the mind.

*Dissection.* The *left* frontal region of the head was much less salient than the right—while the posterior or sincipital region of the same side was in an opposite condition. The meninges were infiltrated. The whole of the anterior lobe of the *left* hemisphere was much flattened, and the substance of this lobe softened, in the centre, and in a well defined space. The ventricles were filled with serum, but there was no other appreciable inequality of development or structure in the two sides of the brain or spinal marrow.

The above cases are followed by a statement of several others in which the patients, paralytic from birth, are still living, and consequently the correspondence between the state of the brain and the atrophied members unascertained by dissection.—*Archives.*

---

### 16. ON THE UNMIXED EFFECTS OF MERCURY ON THE SYSTEM. BY DR. MUSGRAVE, OF ANTIGUA.

Dr. Musgrave, who is already well known to the readers of this Journal, having recently spent some time in the intellectual city, was rather astonished at the general belief there, that the more aggravated affections of the bones, periosteum, throat, and skin, formerly regarded as *venereal*, are, in reality, *mercurial*. This opinion being at variance with the results of twelve years' experience in a climate where mercury is extensively employed, Dr. M. has laudably determined to make these results known to the profession, through the medium of our respected northern cotemporary.*

Dr. M. admits, what every practitioner must admit, that, in certain constitutions long charged with the venereal virus, mercury will be found to aggravate the symptoms, which will often be relieved by a substitution of sarsaparilla or the nitric acid. The same observation he applies to phagedenic chancre. He has, therefore, no unreasonable prejudice in favour of mercury. The following query addressed to the Editor of the Edinburgh Journal is sufficiently pertinent.

"Assuming for a moment this question to be one on which you were entirely without bias, and it became an object to you to ascertain beyond dispute any given effect of mercury, whether beneficial or injurious; what part of the world, let me ask, provided all were equally accessible, would you select as the field of observation and enquiry? Would you content yourselves with an investigation instituted by a class of practitioners here, who sparingly, and with hesitation, prescribe its various preparations by the grain and the half grain?—Or, would you not deem it more satisfactory to ascertain the consequences of its employment under a tropical sun, where, setting aside much deplorable *abuse*, even the most cautious among us,—keenly alive as we are to the inconvenience, nay, the absolute danger of its undue accumulation

---

* Since our last, we have learnt that our junior cotemporary of the North, (the Ed. Journal of Medical Science,) has been discontinued. This is the second quarterly medical journal that has ceased to exist since the commencement of the present year. The failure of the Edinburgh Journal has excited much surprise, considering the extended plan of co-operation on which it was formed, and the number of eminent men who were said to have been embarked in the undertaking. We apprehend that no medical Journal can long exist on such a plan. Where the writers' names are concealed, the *honour* or *credit* of the productions is lost to the owners; and no medical journal can long continue to secure talented pens by the mere force of money.

in the system,—are yet frequently compelled by paramount necessity, arising from past experience of the otherwise fatal result, to administer calomel *by the scruple*, or to repeat smaller doses of from five to ten grains at intervals so brief, as to be justified only by the consideration that we decide between two evils, the greater of which is *death?*

"Or,—to put the query in another shape,—Let us suppose that you had strong grounds for conjecturing that some peculiar morbid affection,—it little matters what,—was the offspring of a certain agency, which had hitherto eluded suspicion; the effect in question being universally attributed to causes of a different kind. Would you, in such a case, look for a confirmation of your conjecture, in the more general prevalence of this affection, towards districts where the influence of the suspected cause was only *partial?*—or would you not rather resort to those where you knew it to be in *full* and *extensive* operation?" 39.

The replies to such queries are too obvious to require any notice, but the following conclusion deserves insertion.

"In order, therefore, to establish a conclusive case against them, framed upon admissions thus extorted from themselves, it will be merely necessary for me to show, *first*, that a practitioner, *ceteris paribus*, would see as much mercury exhibited within *one* year in the West Indies as another could possibly do in *five*, whose sphere of observation was limited to Edinburgh and its neighbourhood: and, *secondly*, that, notwithstanding this lavish consumption within the tropics, we, who have laboured there for a series of years, have not only failed to discover those pernicious consequences for which the anti-mercurialists contend, but have had constant occasion to remark, that examples of what is called secondary syphilis are comparatively rare, under circumstances which, had they the shadow of foundation for their suspicions, ought to render such cases as numerous, or nearly so, as the sum of inhabitants in an island." 39.

Dr. Musgrave next proceeds to give a slight sketch of the practice usually pursued in West-Indian diseases, with the view of conveying some idea of the extent to which mercury is there given.

*Fevers.* The works of Chisholm, Johnson, and Armstrong, are averred to be still the standard guides in the West Indies. In the more concentrated forms of fever, Dr. Musgrave's experience leads him to think that the use of mercury, *as a sialagogue*, may be dispensed with, in order to give full scope to sanguineous and intestinal depletion. The purgative medicines, however, must contain a large proportion of calomel. But the practitioner is liable much more frequently to encounter a form of fever which, from its masked character, lulls the patient into a mistaken security—"and this form is, undoubtedly, treated with greatest success by the rapid induction of ptyalism." Dr. M. describes a dangerous epidemic which ravaged the Island of Antigua in the latter part of 1823, in the form of a malignant intermittent. In this epidemic mercury was the mainspring of the treatment. "By its unrivalled power in equalizing the distribution of the blood, the accumulated mass, lying stagnant in the almost palsied organs, was gradually put in motion. These organs consequently resumed their suspended functions—the wonted secretions were again established, and, being copiously poured forth, the system thus became relieved from an overwhelming oppression." Calomel was the preparation principally used—and before salivation could be induced, it was often necessary to give some hundreds of grains—seldom less than one hundred. In Dr. M's practice, a liberal addition of camphor economised the quantum of calomel. These circumstances should be borne in mind, when we come to enquire what were the sequelæ or effects of this large introduction of mercury, in so great a variety of constitutions.

2. *Erysipelas.* This, in Antigua, stands next, in importance, to fever. Dr. M. properly considers it as much a constitutional disease as gout, and as prone to metastasis. The practitioners of the island had ascertained, long before Dr. Musgrave's time, that saturation of the system by means of mercury, was the only effectual remedy. "Ptyalism was the usual companion of convalescence."

3. *Dry Belly-ache.* Dr. Musgrave's opinions and practice in this disease are known to our readers, by a paper in our number for January 1826. The mercurial treatment is that which is trusted to in Antigua.

4. *Liver Complaints.* It is unnecessary to remark on the common practice of exhibiting mercury in this class of diseases.

5. *Tetanus.* In this dangerous complaint, Dr. Musgrave places most reliance on large doses of calomel, in combination with opium and camphor, preceded by copious bleeding. An external application of oil of turpentine and laudanum, when there was a wound, was employed, and turpentine purgatives were exhibited. Dr. M. has been fortunate enough to see numerous recoveries from this terrible malady, by this plan of treatment.

6. In dysentery, rheumatism, and all acute inflammations, whether of the head, chest, or abdomen, which assume a menacing character, calomel and opium were commonly relied upon, with emetic tartar or camphor, or both, after or in conjunction with blood-letting, purgatives, and blisters. To this list may be added those ill-conditioned sores, *not venereal,* to which the slaves are occasionally liable. These are removed by alterative doses of mercury, without which they will baffle every kind of treatment.

It appears that between the years 1814 and 1826, Dr. Musgrave and his partners received from Messrs. Gordon and Graham, druggists in London, one hundred and eleven pounds of calomel, 61½ of blue pill, and 131 pounds of strong mercurial ointment—all of which was expended in their public and private practice in the island. After this, it can hardly be doubted that Dr. M. has had ample experience of the effects of mercury on various constitutions; and he goes on to assert that, in all this time, he has never either seen or heard of a single case where symptoms, bearing the stamp of secondary syphilis, (in the common acceptation of these words,) were even *suspected* to be the offspring of mercury administered for any other disease—that the *only bones* he has ever seen immediately or ultimately affected by the most aggravated cases of mercurial mismanagement, were those of the upper and lower jaw, whose vitality might have been partially destroyed by a process of denudation, caused by extensive sloughing within the mouth, involving their periosteum. The only other bad effect which he has seen, was one instance of paralysis of the portio dura, apparently resulting from thickening of the conduit through which that nerve passes. Here, then, is strong evidence in corroboration of the statements made by Ballingall, Bampfield, Johnson, and other tropical writers, that mercury, *per se*, does not affect the bones in the manner supposed by the new light gentlemen of the Scottish metropolis. Dr. M. goes on to state facts which leave no doubt that the syphilitic poison itself is amply sufficient to affect the bones without any assistance from mercury. These effects of the poison he has had peculiar opportunities of witnessing among young slave girls, who often conceal their complaints till the malady is far advanced.

We strongly recommend a perusal of Dr. Musgrave's paper to all those who have taken up the modern *hydrargyrophobia*, (if we may be allowed to coin a word for the occasion,) and who not only neglect a powerful auxiliary to blood-letting in many acute diseases, but falsely ascribe to it evil effects on the bones and other structures of the body, for which there is no foundation. As for the Edinburgh philosophers, they are beyond the reach of cure. There is always some epidemic insanity raging in medicine, and this is one of them. The hallucination is fast subsiding, and it is useless to apply any thing but the strait-waistcoat to madmen. When the intellect returns, we may reason with some chance of advantage.

---

### 17. OBLITERATION OF THE AORTA.—SCIRRHOUS DISPOSITION.

The Medical Society of Tours has published some memoirs, in one of which is an account of a case, the particulars of which are deserving of record. A woman, 36 years of age, of good constitution, and who had enjoyed good health till the age of 30, then perceived, in the anterior and lower part of the arm, a small, moveable, but not painful tumour, which gradually increased to the size of an egg, when it became very painful. It was extirpated by M. Dupuytren in 1821; but returning again, the operation was repeated by Dubois. The healing of the wound required the *arsenical paste*. In July, 1824, the woman felt shooting pains a little above the old cicatrix, and soon after had some slight fits of coughing, attended with constriction in the chest, but no expectoration. Catamenia continued regular. In October, the cough became violent, the breathing short, with paroxysms of suffocation, diminution of appetite, loss of sleep. Towards the latter end of November, all these symptoms acquired much intensity. On the 9th December she was removed to the Hospital of St. Come. Her appearance differed little from that of health. She had little thirst, no fever, respiration slow, short, and difficult. It was pronounced by M. Bougon that there was cancer of the lungs. The suffocating fits and dry cough increased—pulse calm—abdomen soft and hot—tender. On the 11th January, 1825, the lower extremities appeared to be paralyzed, and the patient died, though still somewhat corpulent.

*Dissection.* A scirrhous swelling above the original scar—similar tubercles in the thyroid gland, and between the muscular fibres of the right thigh—scirrhous depositions in the substance of the heart—between the ribs and pleura—in all points of the lungs, some as large as a small hen's egg. Some of these tumours had the appearance of scirrhus, some of brain. Stony, calcareous masses occupied the mesentery—others of a medullary nature, the omentum. Scirrhous tumours were found beneath the mucous membrane of the stomach, duodenum, small intestines, gall-bladder; likewise in the kidneys, pancreas, and liver, which was gorged with them. In the interior of the cava inferior, a cylinder of a greenish colour and fibrous texture, was found. Below the third lumbar vertebra, the aorta was filled with a cylinder of a yellowish grey colour, in which was observed a small quantity of puriform matter. In the common and iliac arteries was a substance having the character of ancient coagulum. The texture of the vessels themselves was not altered. All the arteries above the obstruction, were free, and of greater size than natural. There were some tubercles found in the head.

M. Velpeau has adduced the above case as one eminently calculated to shew, 1st, The alteration of the fluids in disease—2ndly, The possibility of obliterating the aorta, without producing death of the lower limbs.—3rdly, The general disposition, known by the name of the "cancerous diathesis."—4thly, The origin of cancer from other than inflammatory causes.—*Mr. Churchill, Med. and Phys. Journal.*

We might here ask the question, had the application of arsenical paste to the wound, any share in the development of this tubercular disposition (for we think the term *cancer* is hardly justified) throughout this unfortunate patient?

---

### 18. MR. BELL ON STRICTURES OF THE RECTUM.

Mr. Bell, in his late lectures at the College of Surgeons, as reported in the Lancet, Nos. 205-6, has touched on the subject of strictures in the rectum. He observes that there are *four kinds* of stricture of this gut, which are necessary to be recollected. The *first* is that which takes place in the integuments around the anus, similar to that which takes place around the mouth, sometimes to such an extent that the patient can hardly take in nourishment through a crow quill. The integuments become firm, lose their elasticity, and become so thickened that a probe can hardly be introduced without great suffering.

The *second* kind of stricture is that which is consequent on piles. The distention of the hæmorrhoidal vessels produces inflammation, and inflammation causes a deposit of coagulable lymph, with loss of elasticity and diminution of calibre in the gut, till the smallest bougie cannot be passed in.

The *third* species of stricture is what is meant by common scirrhus, ascertained by the feeling and the locality. In examining a patient with this complaint, the finger always passes up to the first joint before the contraction can be felt. The stricture takes place at the margin of the inner sphincter. It is occasioned, by habitual constipation, where great straining is used, forcing down a fold of the inner membrane of the gut across the sphincter, where it remains, and is felt by the finger.

The *fourth* kind is the scirrho-contracted rectum, for which the third kind is often mistaken. It is a cancerous disease, beginning in the glandular texture of the part, and resembles the scirrhous contraction of the pharynx. There is great danger in interfering rudely with this disease. In one season Mr. Bell met with three fatal cases of scirrhous stricture, from officious meddling—one in the rectum, one in the vagina, and one in the urethra.

But there are other ways in which the passage of the fæces may be obstructed. There is a very common disease consisting of " a bagginess within the rectum," which very much resembles stricture—and a still more common complaint, caused by constipation and straining—and this is a sudden turning or twisting of the gut where it ceases to be called the sigmoid flexure, and commences to be rectum. This twist will take place for days and months, from straining, and produces all the obstruction which true stricture produces.

These strictures of the rectum are to be removed by regulation of the bowels, so that one copious evacuation may be daily procured, and the bowel allowed to be at rest in the interval. Glysters are useful, as clearing the rectum without irritation. Mr. Bell advises small injections of linseed tea to be thrown up at night, and permitted to remain. The third indication is the bougie—but this is not applicable to all cases. " For instance, for stricture in the very verge of the anus, you had better use the *knife*, and that is the only case in which you can use the knife."

Mr. Bell advises lint to be carefully introduced with a probe, so as to form a kind of ball inside of the stricture, by which the rectum may be drawn out a little. When this is done, a circular piece is to be cut away by sweeping the knife round the verge of the anus. The Lecturer passes some severe *strictures* on those authors who recommend indiscriminately the use of the bougie, " without one hint being given to the reader that he may cause his patient's death by it." After candidly detailing a case where he caused the death of the patient by too freely dilating the rectum by a speculum ani, in a young gentleman, Mr. B. makes the following caustic observation on the speculum and bougie folks:—"You must think that they must have studied surgery in a *cutler's shop,* rather than have been considering the nature of the parts on which they are to operate." We have seen good and evil result from the bougie, as from most other measures in medicine and surgery; but we think the following observation is equally as *sweeping,* and consequently as *unjust,* as some of those doctrines on which the lecturer is dealing out his animadversions.

"Now, having this experience (alluding to the young man who was killed by the speculum ani) am I not bound to tell you that the *simple dilatation* of the anus with the speculum *will produce disease,* and that a peritoneal inflammation *will come on from it;* and you will easily conceive what one feels when he hears others recommending the use of the speculum, and that a great deal may be accomplished by bougies."

Surely Mr. Bell's own good sense must tell him that this unqualified language delivered, ex-cathedra—from the very fountain and source of surgery—from under the very portals of Machaon and Podalirius—and what is more than all, from the mouth of Mr. Charles Bell, must proscribe the speculum ani in toto, as well as the bougie—and, in short, put full one hundred pounds per annum out of the purse of that excellent cutler, Mr. Weiss—an ungrateful reward for all Mr. Weiss's "ingenious contrivances." Mr. Bell's *quondam* opposite neighbour in Windmill-street, (Mr. Thompson) will also be a great sufferer from this and some former *strictures* of the Lecturer on bougies.

An accident prevented us from attending this lecture of Mr. Bell's, at the College, and therefore we cannot say whether or not it is fairly reported; but this we will say, that, as it is now put upon record, it is calculated to do some injury as well as good. Bougies are very useful instruments in strictures of various canals; and the mischief which they occasionally produce in rash or unskilful hands, does not fairly authorise the unqualified reprobation which is used in the lecture as here reported.

---

### 19. GENERAL SUBCUTANEOUS EMPHYSEMA.

The following curious case is related by M. Vitry, in the *Archives Generales,* for March of the present year.

*Case.* A child, 26 months old, of good constitution, had a diarrhœa, then constipation, with loss of appetite, sleep, and spirits. On the 22d of December she had severe fever, which confined her to bed. M. Vitry was called in, and applied six leeches to the epigastrium, with lavements and emollient drinks, which relieved the symptoms during the night and next day, when the fever returned with great intensity, accompanied by severe abdominal pains. The pulse was quick but feeble—tongue moist, white at the base and red at the tip—thirst ardent—cough—obstinate constipation. Neither bleeding, lavements, abdominal fomentations, frictions, nor other remedy had any effect in relieving the symptoms, till the twelfth day of the disease, when copious alvine evacuations were procured, followed by a marked remission of the abdominal pains and the fever. But with this amelioration of the abdominal affection, the cough increased, so as to become constant; accompanied by extreme agitation, and afterwards intense fever, indicating acute bronchitis. Leeches were now applied to the line of the trachea and bronchia, but without any relief.

On the 5th of January, to the foregoing symptoms were added considerable dyspnœa, especially when placed on the left side—face pallid, except the right cheek—thirst urgent. Six leeches to the intercostal spaces under the right axilla, without relief. *6th*, The child had a most tremendous convulsive fit of coughing, soon after which, a swelling appeared at the upper part of the chest, which spread to the neck and face. The tumefaction was evidently emphysematous, and attributed by M. Vitry, to the bursting of some of the air-cells during the cough. The little patient's state became more and more alarming—the restlessness extreme—the sense of suffocation imminent—the extremities cold. During the *7th* and *8th*, the emphysema made progress, invading the whole sub-cutaneous cellular tissue of the head, thorax, and abdomen. The swelling was so great, that M. Vitry despaired of its absorption, and therefore he determined to make some incisions for facilitating the escape of the air. In consultation with several physicians of Versailles, two incisions were made between the ribs, and towards the sternum on each side of the chest, when air was evolved in great quantity. In four hours afterwards the little patient was greatly relieved ; but on the *10th*, two other incisions were made, and the air was freely discharged. From this time, the cough diminished and the child rapidly recovered.

### 20. STENOCARDIA, OR ANGINA PECTORIS.

The following, with some observations, will be found in the Repertor. de Med. of Turin.

*Case.* N. de Volpedo, aged about 60 years, of robust constitution and active disposition, had been affected with several attacks of thoracic inflammations, as well as inflammations of the throat. Towards the end of August 1826, he consulted Dr. Ricotti for an affection of the chest, said to be of five or six months' standing. This came on in paroxysms every day, of longer or shorter continuance, and was considered to be attacks of angina pectoris. The accessions commenced with pain in a single point of the left side of the chest, which soon spread over the whole cardiac region, accompanied by a sense of tightness and oppression in the heart, shortness of breath, acute pains in both arms, and numbness in his hands. The least motion of the body or limbs greatly augmented these symptoms, and he was momentarily threatened with suffocation and syncope. There was a great wish, in these paroxysms, for fresh air. These attacks came on several times a day—and he seldom escaped them for two or three days in succession. They lasted from 20 to 30 minutes, and left the patient suddenly in a state of tremor about the heart, with numbness of the fingers, which went off in a few minutes. In the intervals the patient was perfectly well, all the functions being regularly performed. The paroxysms were easily brought on by any emotion of the mind, whether of a joyous or melancholy nature, or by sudden changes of temperature in the atmosphere. Various physicians were consulted, and all appeared to view the disease as a spasm of the heart, and recommended repose and antispasmodics. Every thing, however, proved useless, and in one of these paroxysms he expired on the 8th September, 1826.

On dissection, the heart was found flaccid and pale, lying in the cavity of the pericardium like an empty bag. There was a white spot on its anterior surface, near the apex. The lungs were gorged with blood of a blue colour. The liver was greatly enlarged, pressing up the diaphragm, and occupying a large portion of the chest.

M. Ricotti attributes the whole of the phenomena, in this distressing case, to the mechanical impediment offered to the heart's action by the encroaching liver. But this is unworthy of a word of refutation. The flaccid and almost rotten state of the heart itself is the true cause of the phenomena in this direful malady.

---

### 21. INFILTRATION OF THE LABIA PUDENDI.

[Dr. W. P. Dewees.]

Among the variety of accidents consequent on parturition, this is neither the least common, nor the least formidable. It is a sudden and excessive distention of the labia pudendi or only one of them, with blood, either during the progress, or very quickly after the birth of the child. It is generally confined to one labium—and, in Dr. Dewees's practice, it has always occurred *after* the birth. Mr. Burns thought the accident was owing to the rupture of a vessel within the nympha; but our author is of opinion that the blood comes from vessels within the vagina, as the quantity is sometimes very large indeed—viz. to the amount of five pounds or more. The vessels, therefore, which furnish such a quantity, and so suddenly, must be of considerable size. In Dr. D's cases, the formation of the swellings required but a very few minutes for completion. Sometimes, however, the formation is slow. This complaint has been mistaken for the distended and protruded membranes, and also for hernia; but a careful examination will easily detect the true nature of the case. Its colour and position are different from those of the above-mentioned conditions. Its position is lateral—unless where the infiltration is double, and then there will be a sulcus between—its colour is that of extreme lividity, or entirely black, resembling neither the membranes nor hernia.

Owing to the unequal density of the external covering and internal face of the labium, the latter becomes irregularly distended, and scarcely any thing is seen but its excessively stretched internal surface. The internal lining of the labium sometimes gives way, and a quantity of fluid or coagulated blood escapes, thus diminishing the extreme anguish of the patient. In all cases there is great pain—in some, it is so severe as to cause syncope. If the bursting do not take place, in the first instance, the tumour is sure to yield, in a short time, from gangrene. When the part sloughs, it exposes a large surface of coagulated blood, which quickly becomes decomposed, and yields an intolerable stench. If the parts do not give way, the pain is agonizing—fever of an active kind is soon kindled up—delirium takes place, and the woman's life is endangered. Her sufferings are augmented by the retention of urine, and nothing but artificial relief can be relied on.

When fever attends, general blood-letting is necessary—and a free incision should be made into the tumour to give vent to the extravasated blood. This should be done without waiting for the process of ulceration, or the chance of bursting. The urine should then be drawn off, by pressing the enlarged labium to one side, and introducing the catheter if necessary. Purgation and the usual antiphlogistic measures must, of course, be added to the above means. A charcoal poultice will help to prevent the inconvenience of the fetor, and lotions of dilute solution of chloruret of lime will be very useful.—*Philad. Journal, May*, 1827.

---

22. HOMŒOPATHY.*

As there is said to be a time for all things, probably the present time is one when we ought to cry, rather than laugh, seeing the profession torn with dissentions and contentions from the South Foreland to the Pentland Firth—the patrons and the professors of Universities at drawn-daggers—and what is far worse, at *law* with one another—physicians and surgeons at war with, and warred on by their respective Colleges, and carrying their petitions and litigations into Parliament and Courts of Justice—one class of the profession endeavouring to supplant another —in short, the contest carried down to individuals, so that in every town, and almost in every street, the foot of one man is opposed to that of his neighbour, while the press inflames the ire of contending parties, and ever and anon cries out—" war even to the knife!"

In such a state of things, it is charitable in our venerable cotemporary of the North, to place before the public a German Farce which, unlike the apple of discord, must raise one universal and harmonious laugh among all ranks of the profession. Our cotemporary has acted on a celebrated precept of antiquity, laid down by no less a personage than Homer himself, and put on record by the wily Ulysses:—

" Hear me my friends who this good banquet grace,
'Tis sweet *to play the fool*, in time and place."

From what has been observed above, we think there cannot be a better time or place, " to play the fool," than the present; and sincerely do we hope that grim-visaged war may smooth his angry front, and " chain the red dragons of his iron car," while attending to the entertainment which our humourous friend, Mr. Spry, has provided for the occasion!

---

* Mr. Spry, Ed. Journal, July 1827.

The scene of the Homœopathic farce, is, as it ought to be, laid in Germany—that huge protuberance of *ideality* in the brain of Europe; and the dramatis personæ are *Professor* Hahnemann and his disciples. It may not be improper to remind the audience, that the principal actor, viz.: the said Professor, "for a long time prepared his own medicines, *to prevent their being known*, and was eventually expelled from Leipzig for thus encroaching on the privileges of the apothecaries, and for persisting to keep his remedies a secret."* The Professor has therefore been obliged to pitch his tent on a kind of No-man's-land, "situated between Magdeburg and the Saxon frontier," where he carrries on an extensive, and no doubt lucrative practice.

The gist of the Homœopathic system may be easily and briefly stated. Hippocrates broached the fanciful doctrine, that a disease should be cured by things that induced a state opposite to that of the disease:—"contraria contrariis medentur." The German Professor strikes out into a diametrically opposite path, and maintains that disordered actions in the human body are to be cured by inducing other disordered actions of the *same kind*, but only much slighter in degree:—"*similia similibus curantur.*" The doctrine of *antipathy* had much foundation both in reason and fact. Thus the burning heat of fever naturally suggests cooling drink and cool air—constipation calls for purgatives—diarrhœa for astringents—soporose diseases demand irritants—irritation calls for sedatives, &c. But what shall we say to Homœopathy? Do venesection and purgation induce diseases resembling pneumonia, ophthalmia, hepatitis, and other inflammations, when these are cured by the above means? The idea is preposterous. But the effects which he attributes to medicines in the most inconceivably minute doses would stagger the credence of any but a German! Thus camomile flowers produce 1481 symptoms—elder flowers 116—iron 228—bark 469—and platinum 402!!! The *thousandth part of a grain* of arsenic is the largest dose that should be given—and the *hundred thousandth part* of a grain is enough in ordinary cases!—"A *drop* of the spirituous tincture of sarsaparilla is said to be a strong dose." The *seven millionth part of a grain of cucumis colocynthis* acts sometimes too powerfully!

It is no wonder that the Leipzig apothecaries chased Hahnemann out of their territory. We understand that a certain WORSHIPFUL COMPANY, in this country, have instituted an action against Mr. Spry, for disseminating a doctrine subversive of the *best interests* of SOCIETY—at least of *their* society. In *this* prosecution, we apprehend there will be no lack of alacrity on the part of the plaintiffs.

* The Charters of the German Colleges seem less favourable to quackery than that which was granted by the amiable (or rather *amorous*) Henry the Eighth of this Country. In Germany, *Quacks* are driven out of society, and regular Graduates of regular Universities are respected. *Here*, the Quack is protected in the exercise of his *art*, but the *Physician* who has undergone the discipline of the first Medical Universities in Europe, is persecuted and *prosecuted* with the utmost rigour of the law!!

### 23. CIRCULATION OF THE BLOOD.*

Neither of these Memoirs has been as yet published to the world in a separately purchaseable form, although both are in print. The first was read before the Philomatic Society in March last, and is inserted in the June Number of "Les Annales des Sciences Naturelles." Dr. Edwards, sen. and M. Jules Cloquet, were named as a Committee, to examine this Memoir, and to witness a repetition of the experiments. Their highly interesting report procured for our author the honour of being named a member of that distinguished society. The second Memoir was presented to the Faculty of Medicine of Paris, on the occasion of the author being received by that learned body, as one of their number. A few copies only have been printed.

We notice these Memoirs with pleasure, because they form a continuation of the Experimental Researches already published in this country, and because they afford us an opportunity of again recurring to a subject, which has of late excited so much interest,—the circulation of the blood.

Dr. Barry's object in the first Memoir is to show, that the tendency to a thoracic vacuum during inspiration, and the consequent suction-effect upon the blood of the veins, are to be found in all the vertebrated animals, as well as in the mammalia. For this purpose, some very curious experiments were performed upon birds, frogs, and fishes, amongst others the following, upon a pigeon. This experiment we, ourselves, have seen Dr. Barry repeat at his lectures.

A tube immersed in a coloured liquid, was made to communicate with the pericardium of the bird.—At each inspiration the liquid rose two centimetres, measured by M. Savant, in presence of the celebrated natural philosopher, M. Ampére. When the trachea was slightly pressed the liquid rose higher and higher, as the pressure was increased, and when the trachea was obliterated, the liquid rose six inches in the tube. The reporters consider the truth of the principles advanced by Dr. Barry in the Memoir, as fully established; and Dr. Edwards thinks that they are likely to exercise a most important influence upon the pathology of the diseases of the thoracic viscera, when accompanied, or preceded by narrowing of the air-passages.

In the second Memoir, (which we are sorry to say can hardly be abridged satisfactorily to the reader,) the author endeavours to prove that the intermitting diminution of the volume of the ventricular heart, by causing a sudden vacuum around its apex, is the cause of its loco-

---

* 1. Memoire sur l'Application du Barometre, &c.—A Memoir on the Application of the Barometer to the Study of the Circulation of the Blood, and of Respiration, in the vertebrated Animals.

2. Dissertation sur le Passage du Sang, &c.—A Dissertation on the Passage of the Blood through the Heart.

By D. Barry, M. D. of the University of France;—Member of the Faculty of Medicine of Paris, of the Royal College of Physicians of London, of the Philomathic Society, and of the Society of Natural History of Paris, &c. &c. &c.

motion or stroke, and consequently of the rapid filling of the appendices at its base.

2*ndly*, That the constant efforts of the heart to contract its cavities, produce the commencement both of inspiration and expiration in the mammalia. The former being the pressure of the atmosphere down the trachea to obliterate the cavities around the heart, the latter the same pressure acting upon the outside of the animal, for the same purpose. Contractility alone is thus made the agent of the circulation, both of the air and of the blood.

A very extraordinary experiment is detailed in this Memoir, which from want of space, we regret we cannot give at full length. The hand and arm were introduced into the thorax of a living horse, in such a manner as that no air was admitted. The vena cava was grasped between the diaphragm and the heart, and the blood was felt distinctly to fill the vein, and pass on in a copious current to the auricle, *only during inspiration;* the vein becoming collapsed and empty during expiration. The appendix only, and not the whole auricle was found to alternate in its contraction and expansion, with the ventricles.

From this latter circumstance, the author is led to conclude, that the sounds produced by the action of the heart, (as heard through the stethoscope,) are the results of the dilatation of the cavities of that organ, and not, as stated by M. Laennec, of their contraction. The first sound is that of the dilatation of the two appendices. The second of the two ventricles.

As we understand that Dr. Barry means shortly to publish a detailed account of these experiments, and of his own views as to their results, we shall abstain from going further into particulars at present. We must, however, warn such of our readers as are opposed to this gentleman's theory of the circulation and absorption, that he takes the field for the next campaign, with a very considerable addition indeed, to the force which he has hitherto shown. That truly philosophic and acute observer, Dr. Edwards, the author of "The Influence of Physical Agents upon Life," has declared himself, in the most unequivocal manner, on Dr. Barry's side of the question. This name is in itself a host. The Prize Committee of the Medical Society of the Northern Athens has, we understand, adjudged their prize this year for the best Essay on Absorption, to our author. Dr. Carson of Liverpool, after a long absence from the polemic arena of physiology, now leads boldly up to Dr. Barry's assistance, his pulmonary resiliency, and self-expanding ventricles and auricles. He has already come to blows with the enemy, and brandishes in their very ranks, the *lancet* and the *yellow flag**. Though his dogmas differ from those of Dr. Barry, the main point for which they both contend, is the same.—To prove a suction-effect exercised upon the blood of the veins.

That the thorax, by its expansion acts as a pump, it would be absurd to deny. That this pump influences liquids in communication

---

* Vide London Medical and Physical Journal for August.

with the great thoracic veins, under certain circumstances, all allow. In expressing ourselves convinced of this, in a former number, we lamented the difficulty of ascertaining the amount of the suction-influence. This difficulty, we think Dr. Barry has in some measure surmounted, by the application of the barometer. He has, at least, shown that the opposition to the entrance of the blood into the chest is considerably lessened during inspiration. We have no reason to suppose that an animal will make greater efforts to expand his thorax, after a pointed tube has been plunged into it, than before. On the contrary, the wounded animal evidently restrains these efforts. Yet a pigeon, under these circumstances, lifts water one inch; a small dog six or eight inches; a horse double this at least. We are much under the results obtained by Dr. Barry. But supposing the suction-power to be as we have stated, Dr. Arnott's "bloodless experiment" must involve one or more sources of error. He held in his mouth a tube, one end of which was immersed in water, whilst he breathed through his nostrils. He states, that though "the mouth may, under these circumstances, be considered to form a part of the cavity of the thorax," the liquid scarcely rose at all in the tube during inspiration. This result we should have expected; 1st, because the *velum pendulum palati* being applied closely to the root of the tongue when we breathe through the nostrils, cuts off all communication with the trachea. 2ndly, Because the mouth never forms a part of the bag of either pleura, nor of the pericardium, nor of the cavities of the great veins under any natural circumstances. Though, as we have stated in a former number, we fully agree with Dr. Arnott in his views of the *arterial circulation*, we cannot subscribe to his reasonings as to the venous circulation and absorption. Indeed, we have little doubt, that upon reflection he will himself perceive, how untenable some of his arguments on these subjects are.

He says that no pump could suck liquid through the veins, because they are compressible tubes "and free to collapse." Now surely Dr. Arnott, or any other man that reads his very able work, nay any man of common sense, who knows that the veins are kept constantly full to the level, at least of the diaphragm, must see, that a pump acting at this point, would raise the liquid thus sent up by a vis a tergo, with much more ease through the veins, than through incompressible tubes of the same length. Dr. A. it appears, can no more do without a pumping-power in his plan of the circulation, than Drs. Barry and Carson. This notion is as old as Pecquet. In his day the heart was called "*vitalis antlia*,"* the pump of life. Dr. Carson added the resiliency of the lungs as another pumping-power. Dr. Barry makes the whole chest a pump—Dr. Arnott makes each capillary a pump. They "(the capillaries,) work like innumerable little pumps, emptying the arteries into the veins"† even after death. This, as Jonathan says, would be

* Vide Pecquet Dissert. Anat. de Circulatione sanguinie et Chyle Metu (p. 111.)

† Vide Elements of Physics (p. 498.)

"important if true." But in attempting to follow Dr. A. in his logic as to the functions of the capillaries and the absorbents, we saw such conclusions in the perspective, and such routes leading to them, as must frighten men of plain understanding like ourselves. There is still much to be learned in hydraulics. The article on this subject, in Dr. A's. book, if it is to be applied to the circulation of the blood with useful effect, must certainly be written over again. We consider this section the poorest in his very interesting book. The author has not, in our mind, established by a single proof the views which may be said to be his own, nor has he overthrown those of Dr. Barry. There is an apparent soundness and simplicity in this latter gentleman's arguments, a kind of irresistible conviction in his reasonings, which the mind, (having once admitted the principle,) cannot refuse to follow. "The elegant rotundity" of this theory (to use the phrase of a northern contemporary,) is perfectly unassailable, except at one point, the fundamental experiment, or rather the principle upon which that experiment is based. This point of his trenches, however, the military Dr. appears to have strengthened considerably, by extending his works into all the classes of the vertebrata, and by measuring the suction-power in each. Like the present system of astronomy, which supposes the earth to move round the sun, instead of the sun moving round the earth, this theory explains every thing explainable by the old doctrines, and many other things besides, which, according to these doctrines, appeared absurd, if not impossible. The respiratory pulsation of the external jugulars—the rising and falling of the exposed brain—the modified hæmorrhage even from distant capillaries, as respiration becomes deep, prolonged, or restrained—gaping, or expanding the thorax to its utmost capacity, when we are fatigued, or exhausted—death by sucking air into the heart, through a wounded vein—the rapidity with which a poison is absorbed through the coats of a vein, compared to those of an artery—the beautiful and important effect of the cupping-glass, in preventing, or arresting this absorption—the venous reservoirs of diving animals—these and a thousand other phenomena perfectly inexplicable by, and apparently unconnected with, all that we hitherto knew of the circulation, are now amongst the most simple and intelligible physiological facts. What is there satisfying to the mind in the following assertion: "Absorption is a strong action of life."* What does this proposition help to explain? What induction does it lead to? How is it demonstrated? It is not even what M. Magendie has termed it, "the pure and simple expression of our utter ignorance on the subject?" It is, in the present state of our knowledge, an awkward, uncandid, illogical attempt at concealing our ignorance. We know from Haller, and from experiment, that absorption goes on long after death. "Quietis tamen intestinis, et multa morte frigidis, chy-

* Vide Elements of Physics, &c. (p. 513.)

lum moveri et effugere."* Absorption, therefore, cannot be a *strong* action of life abstractedly considered; for we see that it takes place after life has ceased.

We cannot close this article without expressing a hope, that Dr. Arnott may be induced to employ his knowledge of physics in direct experiments upon the living animal. Let not the Dr. be alarmed. "Bloodless experiments," will not always do. We quote from an authority, in bowing to whom, he "need not blush."† It is evident that there are, in the living machine, mechanical agents that have entirely escaped his contemplation. Such for instance as the power which the heart possesses, of diminishing its own volume, within a shut and incompressible cavity. This important fact is proved by Dr. Barry's last experiments upon the dead horse; the living frog, &c. One practical, well established fact is worth a cart-load of theoretical speculations.

We are sorry that we cannot refer our readers directly, to the very remarkable Memoirs which we have been reviewing; but we trust Dr. Barry will speedily enable us to do so, by giving them to the profession at large, in a more detailed form.

### 24. PERFORATION OF THE BLADDER.

Mr. Serph, surgeon, of Welch Pool, has published a curious case, in respect to the final treatment of which, he solicits the advice or suggestions of his brethren. We shall give a brief abstract of this case, referring our readers to the original communication, in our contemporary of the present month.‡

*Case.* Mrs. A. aged 35, had enjoyed good health till the month of January, 1823, when she was seized with severe pains in the loins, lasting 14 days. In the following May, the patient was attacked with acute pain in both hip-joints, which confined her to bed for a week, and rendered her lame for a few weeks afterwards. In August of the same year, 1823, she discovered, for the first time, a small, deeply-seated, but loose tumour, in the left labium pudendi, which increased, without pain, and burst, in August, 1824, discharging scrofulous matter. A fistulous opening has remained ever since, from which there constantly oozes some fluid. In March, 1826, Mrs. A. began to feel irritation in the bladder, and, in November of the same year, a calculus was discovered. On the 29*th*, Mr. S. introduced Weiss's dilator, and, in 20 minutes, dilated the urethra sufficiently to be able to ascertain, with his finger, the position of the stone. A common dressing forceps removed the calculus, which was of the mulberry kind, and the size of a peach-stone. The bladder never for an instant lost its retentive power, but it remained very irrita-

* Haller's Element. Phys. lib. 25, vol. vii.

† Vide Bacon de Augmentis Scientiarum.

‡ Med. and Phys. Journal, for October, 1827.

ble, and the patient complained of a constant and fixed pain in the lower part of the abdomen on the left side. There now came on a discharge of muco-purulent matter (very fetid) from the bladder, indicating ulceration of that viscus, for which various remedies were employed, without any good effect. The symptoms of irritation increasing, the patient became convinced that she had another stone in the bladder, and this conviction was verified; for, on the 7th May, 1827, Mr. S. proceeded to a second operation, which was very tedious, in consequence of the friability of the calculus, which crumbled between the fingers, like half-dried mortar. All the particles being washed away, the surgeon and patient now flattered themselves that they had conquered the enemy; but this was not the case, for the pain in the left side of the bladder returned, and early in July, the afflicted patient again asserted that a calculus still remained in the bladder. A third operation was performed on the 14th July, and the bladder was examined, but no stone could be found. Mr. S. was on the point of giving up the search, when, turning the finger towards the posterior surface of the pubis, he detected a rough substance, part of which crumbled under the touch. Mr. S. endeavoured to loosen the remainder, which was so rough and so hard, that he was obliged to desist, on account of the pain it gave him. He then introduced a lever, curved to an angle of 45°, and twice fixed it on the hard substance, and "applied to it a force much more than sufficient to break it in pieces, had it been of a consistency even much harder than urinary calculi generally are; but all in vain." In this attempt, "a piece of irregular shape broke off, which was of a dark colour, and extremely hard." The finger was introduced, and the substance "felt of that kind of looseness, which we sometimes feel in shaking between the finger and thumb some of the molares, which, however, require a great power to extract them."

It appears to Mr. S. that, so long ago as 1823, the disease began to develop itself in the os pubis of the left side, and that that bone became carious—secondly, that an abscess formed, which interested externally the labium of that side, and found its way internally through the coats of the bladder—thirdly, that an osseous excrescence, arising from the pubis, penetrated through that opening, and became a nucleus, on the surface of which, the calcareous part of the urine was partially deposited, in the same way, as stalactites are formed—fourthly, that, when the concretion had acquired a certain size, it detached itself, and became a common urinary calculus, loose in the bladder—fifthly, that, had he succeeded in breaking off the bony process, some accident might have arisen from the contact of the urine with the sound part of the bone.

The writer, as we before observed, solicits the opinion and advice of his professional brethren respecting the prognosis and treatment of this remarkable case. At present, the patient is taking iodine, apparently with advantage.

---

### 25. CANCER OF THE CARDIA.

A lady, aged about 40, became affected in the Month of November, 1826, with common symptoms of dyspepsia, and her complaint was considered as such by her medical attendants. In the beginning of 1827, the patient began to complain of some difficulty in swallowing solid food, and sense of nausea at the stomach after meals. These symptoms gradually increased to inability to swallow food, or rejection of the food undigested a few minutes after it was taken, by a species of regurgitation rather than vomiting. Mr. Brodie, Dr. Johnson, Mr. Andrews and others were consulted, and the case was considered to be disease about the cardiac orifice of the stomach. Blisters, local bleeding, and various means were used, without arresting, in the slightest degree, the rapid progress of the disease. During the month of May, very little food could be got into the stomach, and constant pain was complained of in the region of the cardia. The patient was nourished principally by enemata. In the beginning of June, she spat up rather suddenly a bloody kind of offensive matter, and all at once she acquired power, not only of swallowing, but of retaining the food. She now took a good deal of nourishment, and some faint hopes were entertained of recovery. But the discharge from the œsophagus increased in quantity, and became deteriorated in quality—the fever wasted the patient—the thirst was constant—bad matters were passed by stool—the stomach again rejected food, and death put an end to her sufferings, towards the end of June. The dissection was carefully made by Mr. Howship, in the presence of Dr. Johnson, Mr. Andrews, and Mr. Carrick of Kensington. The lower portion of œsophagus, where it enters into the stomach, was in a state of open or ulcerated cancer, for the space of about three inches. A communication had taken place with the right cavity of the chest, in which was found some sanious effusion, and a considerable portion of lung contiguous to the original disease was disorganised and broken down. The stomach itself, and intestines were sound.

This disease was remarkable for the rapidity of its growth. Nine months previous to its fatal termination, the lady was in perfect health. In this respect, disease of the cardiac orifice of the stomach forms a striking contrast with that which affects the pyloric orifice. Where pyloric disease takes place, the food gets readily enough into the stomach—digestion goes on—and some chyme passes into the duodenum till the last. Emaciation is slow and progressive, and vomiting is a pretty constant symptom some hours after the food is taken into the stomach. Where the cardia takes on disease, on the other hand, the food is, in a great measure, prevented from getting into the stomach, and is regurgitated rather than vomited. The system is deprived of nutriment, and death soon closes the scene.

### 26. OMISSION OF LIGATURE IN AMPUTATION.

[Dr. Koch, of Munich.]

Notwithstanding the researches and experiments of surgeons and physiologists, respecting the spontaneous cessation of hæmorrhage from divided vessels, much uncertainty and much contradictory opinions still remain. The author of this paper thinks that *timidity* has tended to keep us in ignorance on some points of importance. There are very few who will amputate a limb, and fearlessly trust to nature for the security of the cut vessels. *The author's father, Director of the General Hospital of Munich, has not tied a single artery in the various amputations which he has performed for the last twenty years.* To this wide range of experience, the son has added his own, in corroboration of the opinions of his father and of himself, respecting the imaginary danger of leaving vessels untied in amputations.

Arteries, says he, when cut and not tied, remain entirely open, up to the place where they are divided:—The canal of arteries tied in the usual manner remains open also to the spot where the ligature is applied, and their parietes do not unite at this spot. These observations were repeatedly made by the author's father, on dead bodies, where the arteries had been cut by him, or tied by other surgeons, many years previously. He always found the diameter of the vessels that had *not* been tied, contracted as they approached the place of section, but the parietes never adherent till the artery ended in a kind of cicatrix. These things are seen in numerous preparations by the author, in the anatomical museum of Munich. The vessels that had been tied presented the same appearances, except that, at the spot where the thread had been applied, there was a narrowing, but never an obliteration of the canal of the vessel.

In a disarticulation of the hand, the surgeon had tied the radial artery, and omitted to tie the ulnar, as it did not bleed. The ligature came away on the 8th day, and on the succeeding day the patient died. On examination, the terminations of the two arteries were so similar, that it was difficult to say which of them had been tied by ligature. Both extremities were perfectly pervious—the radial artery appeared to be slightly torn at the termination.

In numerous experiments on dogs, our author could perceive no difference between the arteries that had been tied, and those that were left to nature. Thus, he tied the femoral artery of a dog, and cut the vessel below the ligature, without hæmorrhage. The wound was closed and healed. A month afterwards he killed the animal, and found the upper and lower extremities of the vessel were completely similar, each being united to an external coagulum by an open mouth.

The formation of a coagulum takes place in some, but not in all cases. But it produces the same effects in the vessels which are tied, and in those which are cut, and not tied. In ligatures of arteries, the internal coagulum is often found in connexion with the external, so as to fill exactly the orifice of the vessel. The coagulum is rarely adherent to the internal parietes of the vessel, and never completely so up to the nearest anastomosis. An amputation was performed at the hip-joint, and the crural artery was tied. The ligature came away on the eleventh day, and, on the fourteenth day, the patient died. On dissection, a clot was found plugging the artery, but the canal of the vessel was open. In a very few cases indeed was the bore of the artery found obliterated after the ligature, by adhesion of the sides of the vessel.

"No doubt," says the author, "that most surgeons will stare when I propose the general abandonment of the ligature, as the means of preventing hæmorrhage, especially in amputations. They will be still more surprised when I assert, that, by this omission of the ligature, the most certain means are taken to obviate effusion of blood. Yet this assertion rests on the basis of experience, and can be testified to by all those who have witnessed my father's operations in a public hospital for twenty years past."

Dr. K. appears to think that the spontaneous cessation of hæmorrhage from a divided vessel depends *chiefly* on some change in the blood itself —partly in retraction of the vessel. The coagulum he considers as the *effect* rather than the *cause* of this cessation of hæmorrhage. This last conclusion appears plausible; for it is hard to conceive that coagulum can form *during* hæmorrhage—and if it form after the cessation of the flow of blood through the orifice of the vessel, it can hardly be viewed in the light of a *cause* of that cessation. All that can be said in this case is, that the coagulum may prevent subsequent hæmorrhage; but this our author denies.

"The application of the ligature," says he, "in disturbing the spontaneous cessation of the hæmorrhage, acts in a manner quite opposed to the end in view. It produces, it is true, a mechanical and temporary obliteration of the bore of the artery, but this is inferior in value to the natural retraction of the vessel, and spontaneous cessation of the hæmorrhage."

This spontaneous cessation is to be aided, or rather promoted, by pressure on the trunk of the vessel leading to the part, and a gentle degree of the same on the face of the stump, either by the hand or by a proper bandage. By these means the stasis of the blood is promoted, and protection from future hæmorrhage secured.

The method pursued by Dr. K. and his father in amputations is as follows:—After dividing the soft parts and bone, the surface is sponged, and the muscles and integuments brought neatly into contact, and retained by adhesive plaster, so as to secure adhesion by the first intention, if possible. *During* the operation, the vessel is compressed by the fingers of an assistant, and *afterwards*, the pressure of the fingers is rendered unnecessary by the application of a compress, laid along the trajet of the main artery, secured by a roller. The patient is then placed in his bed, and the stump kept elevated, and an assistant is directed to make gentle pressure on the face of the stump for an hour or two—or longer, if he feel considerable pulsation in the part. "When this pulsation has ceased, and when the dressings appear tinged red by the exuding lymph, all danger of hæmorrhage is considered as at an end, provided the patient keeps quiet. Presently, the exudation of lymph ceases —and the dressings become quite dry and cold." The patient generally passes the first few days without fever, on which account he is allowed wine, coffee, and other food, which dare not be given under other circumstances. No opiates or medicines of any kind are usually exhibited after the operation. About the fifth day, there is generally some traumatic pyrexia evinced, owing to the suppurative process going forward in the wound; but it requires no particular treatment. A moisture taking place on the dressings about the seventh day, indicates the establishment of suppuration; but if the dressings keep dry, union by the first intention is sure to have occurred. Whether suppuration or adhesion has taken place, the dressings are never removed before the tenth day, or even later, unless violent inflammation or hæmorrhage should arise. They consider that the adhesion of the integuments and muscles is never properly consolidated before the tenth or twelfth day, and, therefore, that much mischief is done by too early a removal of the dressings.—*Journal de Progrès.*

We leave these statements supported as they are by occurrences in a public hospital, to the consideration of surgeons and physiologists. They are worthy of consideration.

---

### 27. CONGENITAL ENLARGEMENT OF THE HEART.

Mr. J. was born in 1811, and was a sickly and feeble infant. When very young, he evinced a laborious state of breathing, and impeded functions of the heart and lungs. He spoke with volubility, but was often obliged to stop, in the midst of his speeches, to take in breath. His lips were habitually of a blue colour, as well as the extremity of the nose, and the ends of the fingers. If he took any brisk exercise, he was soon panting for breath, and his heart in violent action. In this state he grew up—always in the doctor's hands—but never cured. It appears that he had several illnesses of an inflammatory character, for which he was bled, blistered, leeched, &c. It was observed that the *right* side of the chest was flattened in, and the *left* bulged out in the region of the heart. The left hypochondrium was also prominent. In short, it was evident that the functions of respiration and circulation were greatly disordered, but whether the structure of the heart and lungs was affected, was the question. At the age of puberty, all the symptoms above-mentioned became greatly exasperated, and in spite of various remedies, death put an end to the patient's sufferings.

*Dissection.* The left side of the chest seemed bulged out in the region of the heart, while the right side seemed depressed. The left arm was emaciated, the right was swelled. On opening the chest, they were astonished at the size of the heart. It occupied, in a great measure, both sides of the thorax! The pericardium contained about three ounces of water. The right auricle was prodigiously dilated, and its parietes attenuated. It contained a large quantity of coagulated blood, disposed in layers, and intermixed with fibrinous concretions. The pulmonary artery was dilated from its origin to its division into two branches, the right one of which was also dilated, while the left pulmonary artery was diminished, so that it would scarcely admit a probe. The ductus arteriosus was open, as in the fœtus, so as to give free communication between the pulmonary artery and the aorta. The foramen ovale was closed. The right ventricle of the heart was dilated and attenuated. The left ventricle and auricle were also greatly enlarged, but their parietes were proportionally thickened. What lung was left occupied the right side of the chest, and was gorged with blood. The left lung was reduced to almost nothing, and evidently performed no respiratory function. There was some effusion in the abdomen, and the liver was large, and gorged with blood. The vena portæ and the vena cava were varicose.

The narrator of the case (M. Cogoreux) thinks, and with great probability, that the heart was originally too large, and, consequently, pressed on, and ultimately annihilated, the left lung—hence the enlargement of the pulmonary artery of the right lung. We see, in this case, two opposite conditions in the two sides of the heart. The right chambers were in a state of passive—the left, of active aneurism.

### 28. COLLARED CASES IN MIDWIFERY.

[Mr. Smith. Ed. Journal.]

It is by no means uncommon to find the funis twisted round the neck of the fœtus in parturition. We are directed, in such cases, to draw the funis over the child's head; or, if that be impracticable, to divide the navel-string, and secure the fœtal extremity by ligature. The first plan is more easily recommended than practised—and is not always free from hazard of rupture of the cord, or separation of the placenta, with consequent hæmorrhage. The division of the cord is still more dangerous—at least to the child. Mr. Smith's plan is this:—Having waited the usual progression of the head through the pelvis, until it is excluded from the vagina, he first ascertains which is the umbilical, and which the placental portion of the cord, by observing that the fœtal portion will invariably be found tight and unyielding, whilst the placental will easily yield to a slight effort of extension with the fingers. This done, he avails himself of as much of the placental portion as he can obtain, and gently extends it to the extreme points of the shoulders. He then waits for a pain, during which, he forms, with the fingers of both hands, an ellipsis of the funis, of sufficient size to admit the escape of the shoulders and trunk through it. The result is obvious and immediate: the labour experiences no interruption, and the delivery is effected by the fœtus passing through the extended noose of the cord, whilst the fundus uteri remains undisturbed, the placenta undetached, and the string unbroken.

### 29. CASE OF SUPPOSED HYDRO-PERICARDIUM CURED.

Dr. Bonet has offered the following case to the Professional Public. A man, 66 years of age, had been subject, for many years, to pains in the ankles and other joints, which were supposed to be of a gouty nature, as they generally lasted but a few days at a time. For five years, he complained of being easily put out of breath, on ascending stairs, or walking up an ascent, so that he was often obliged to stop short, or even sit down. The quickness of breathing, at those times, was attended with paroxysms of coughing, without expectoration. He also complained of a dull, deep-seated pain under the sternum, in the region of the heart. At these times, his face, naturally pale, would become flushed, and his lips livid. When Dr. B. was consulted, the patient's eyes were yellow, the breathing short, the paroxysms of cough were frequent, with occasional expectoration; there was acute and deep-seated pain in the region of the heart; decubitus lateralis impossible; pulse full, hard, and only 45 in the minute, with an occasional intermission. By the stethoscope, and the naked ear, a loud noise was distinctly heard in the region of the heart, at each contraction of the ventricles. These phenomena induced Dr. B. to conclude that there was water in the pericardium. There was, at this time, no œdema of the extremities; but the symptoms getting worse, the legs began to swell in a week after this period, and the swelling progressively increased till the limbs were double their natural size. Ascites ensued, and paracentesis was contemplated. The œdema gained the body and upper extremities. The patient's state was now hopeless, and he had frequent attacks of syncope. Leeches had been several times applied to the region of the heart, and diuretics had been given internally. These not succeeding, our author determined on drastic purgatives, combined with diuretics. Calomel, jalap, scammony, aloes, and digitalis were combined, so as to produce every second day a purgation of five or six brisk evacuations. This treatment was continued six weeks, with the effect of reducing the dropsical swellings of all parts, the difficulty of breathing, the cough, the irregular action of the heart, the decubitus difficilis, and, in short, all the symptoms of the complaint. The cure has been complete; or, at least, there has been no relapse during the last six months.—*Journ. Complementaire.*

We do not see that there was any very positive evidence of hydro-pericardium in the above case. We think the disease was more of the hepatic than the circulating system, and the success of the treatment justifies this conclusion. Palpitation and irregular action of the heart are often among the first symptoms of dyspepsia and biliary derangement. It is not unlikely, however, that there was some effusion in the chest, at the time when the other dropsical symptoms obtained—and all the dropsical effusions were of an inflammatory character.

---

### 30. MEDICINA FORENSICA.

The following documents will excite considerable interest at the present moment, if taken in connexion with the article which we have dedicated to an examination of the College Charter in the present Number of the Journal:—

CORRESPONDENCE between *Dr. Harrison* and the *Censors* of the *College of Physicians.*

No. I.

*Copy of the Censors' first Communication to Dr. Harrison.*

July 6th, 1827.

We, the Censors of the Royal College of Physicians, London, having received information that you are practising physic within the city of London, and seven miles of the same, do hereby admonish you to desist from so doing, until you shall have been duly examined and licensed thereto, under the common seal of the said College, otherwise it will be the duty of the said College to proceed against you for the recovery of the penalties thereby incurred.

WILLIAM LAMBE,
J. COPE,
H. H. SOUTHEY,
CORNWALLIS HEWETT.

College of Physicians, Pall Mall East.

A board for examining persons who have the requisite qualifications, will be holden at the College on next Friday, 13th July, 1827.

*To Dr. Edward Harrison, Holles Street.*

No. II.

*Copy of Dr. Harrison's first Answer to the Censors' Communication.*

Holles Street, 7th July, 1827.

Gentlemen—I had last night the honour of receiving a communication, purporting to be signed by *you*, as the Censors of the Royal College of Physicians, London, wherein you are pleased to admonish me to desist from practising physic within the city of London, and seven miles of the same, until I shall have been duly examined and licensed thereto, under the common seal of the said College; and alleging that, otherwise it will be the duty of the said College to proceed against me for the recovery of the penalties thereby incurred.

Before I answer the above communication, will you have the goodness to point out to me the authorities under which you act, and the penalties to which you allude?

I have the honour to be, Gentlemen,
Your very obedient humble servant,
EDWARD HARRISON.

*To Drs. Lambe, Cope, Southey, and Hewett,*
Royal College of Physicians, London.

No. III.

2, King's Road, Bedford Row, 9th July, 1827.

Sir—Being senior Censor of the College of Physicians, I opened your letter of the 7th instant, which I shall lay before the Board at their next meeting. I think it right to inform you, that such meeting is appointed for Friday next, at three o'clock, where you may appear, *if you think proper*, and obtain whatever information the Board may think it their duty to communicate to you.

I am, Sir, your obedient servant,

WILLIAM LAMBE.

*Dr. Harrison, Holles Street, Cavendish Square.*

No. IV.

Holles Street, July 12th, 1827.

Sir—I beg to acknowledge the receipt of your letter of Monday last; and I have only to repeat the request made by my former letter.

I have the honour to be, Sir,
Your very obedient humble servant,
EDWARD HARRISON.

*Dr. Lambe, 2, King's Road, Bedford Row.*

No. V.

*Copy of Censors' second Communication to Dr. Harrison.*

College of Physicians, July 19th, 1827.

Sir—We, the Censors of the Royal College of Physicians, London, having taken into consideration the request made in your letter of the

7th July, "to be informed under what authorities we act, and what are the penalties to which we alluded"—have to inform you, that WE ACT UNDER THE AUTHORITY OF OUR CHARTER, CONFIRMED BY PARLIAMENT 14TH AND 15TH HENRY VIII., which is well known, and has been repeatedly inforced.

WILLIAM LAMBE,
CLEMENT HUE, vicarius for Dr. COPE,
H. H. SOUTHEY,
CORNWALLIS HEWETT.

There will be a Censor's Board held at half-past four o'clock on Thursday next, 26th July, at which, *if you think proper*, you will have an opportunity of appearing.

*Dr. Edward Harrison, Holles Street.*

No. VI.

*Copy of Dr. Harrison's second Answer to the Censors' Communication.*

Holles Street, July 23d, 1827.

Gentlemen—After acknowledging the favour of your letter of the 19th instant, informing me "that you act under the authority of your Charter confirmed by Parliament 14th and 15th Henry VIII.;" I beg leave to observe, that I can no where find the title of "Censors of the Royal College of Physicians," mentioned in that statute; or their right to examine Graduates of Universities recognised. And what is no less relevant in this case, it does not appear to confer the power of constituting two different classes of physicians, under the denomination of Fellows and Licentiates. Such is the result of a careful examination of the above-named act. But if you can point out particular clauses in it by which the title and powers in question are distinctly given, I shall feel obliged by the communication.

I have the honour to be, Gentlemen,
Your very obedient humble servant,
EDWARD HARRISON.

*To Drs. Lambe, Cope, Southey, and Hewett,*
College of Physicians, London.

No. VII.

*Copy of Censors' third Communication to Dr. Harrison.*

College of Physicians, July 26, 1827.

We, the Censors of the College of Physicians, have received your letter, bearing the date of July the 23d, 1827, and have nothing to add to our last communication, excepting that the next Censors' Board for examining all persons, *who have the requisite qualifications*, will be held at the College of Physicians on the 1st of next October, at four o'clock, P. M.

WILLIAM LAMBE, H. H. SOUTHEY,
J. COPE, CORNWALLIS HEWETT.

No. VIII.

*Dr. Harrison to the Censors of the College, (delivered August 6th.)*

Holles-street, Aug. 4th, 1827.

Gentlemen,—In reply to your communication, bearing date of the 26th of July, I desire to state that I was led in my last letter to propose three questions for your consideration, with the view of encouraging amicable discussion, and of producing, if possible, a conformity of sentiment between us. As my conciliatory efforts have been frustrated by your uncompromising answer, I beg to inform you, that, having bestowed no inconsiderable attention upon the constitution of the College of Physicians, I am led to conclude, that the privileges and powers granted to it by the statute 14th and 15th of Henry VIII. are not now in existence, or at least are no longer available for College purposes. Upon the consideration generally of the other insurmountable difficulties and objections to the exercise of your monopoly, I shall think it unnecessary to enter, unless the paramount one can be removed.

Intelligent and enlightened physicians, as well as gentlemen learned in the law, entertain similar opinions to my own. I have also reason to know, that even among those who formerly ranked with the highest of the Fellows, the boasted authority of the College was denominated a mere 'Brutum fulmen.' As I had not lately met with opposition from any of the Fellows, in the exercise of my professional duties, I concluded that I should be suffered to pursue them without further molestation,—until I was roused from my deceptive quiet, and forced into the field by your colleague, Dr. Chambers.

Fortified with the concurring approbation of accomplished lawyers and physicians, I thought that I could not bestow greater service upon the medical profession, to which I am enthusiastically devoted, than by bringing all disputed matters formally into open court, under a conviction that, however they may be decided, the interests of the faculty and of the public will be essentially promoted by the investigation.

Actuated by these motives, I have tendered to the College for a series of years, through some of its Fellows, opportunities of examining legally their pretensions to interfere with me, or my practice.

I am unwilling to suppose that the gentlemen who now compose the 'Royal College of Physicians,' and for whom, individually, I entertain every respect, are less desirous than myself to come at once to the points at issue between us, without the introduction of those foreign topics which have hitherto embarrassed the question, and prevented a satisfactory decision.

Acting on public grounds only, and for the advantage of our common profession, I pledge myself that, if proceedings are instituted, they shall on my part be carried on without unnecessary irritation or excitement.

In pursuance of the great objects for which I contend, I now declare, that I do not recognise your authority as 'Censors of the Royal College of Physicians,' and shall therefore decline your invitation to offer myself at your Licensing Board 'on the 1st of next October.'

I have only to add, in concluding my correspondence, that Messrs. Tennant, Harrison, and Tennant, of Gray's-inn, are my solicitors. To them I refer you, in case of your choosing to institute proceedings against me. They are furnished with instructions to give every facility to a legal investigation of your assumed privileges; but they are directed neither to compromise my rights nor those of my professional brethren.

I have the honour to be, Gentlemen,

Your very obedient humble servant,

(Signed) EDWARD HARRISON.

*For Drs. Lambe, Cope, Southey, and Hewett,*
College of Physicians.

